# Dynamic changes and clinical significance of the gut microbiota and serum metabolites in breast cancer onset, progression and chemotherapy intervention

**DOI:** 10.3389/fonc.2026.1795317

**Published:** 2026-05-14

**Authors:** Shuyun Jiang, Zhanwei Du, Yufei Wang, Hongwei Ma, Zhijun Ma, Xiaowang Wang

**Affiliations:** 1 College of Clinical Medicine, Qinghai University, Xining, Qinghai, China; 2 Department of Surgical Oncology, The Affiliated Hospital of Qinghai University, Xining, Qinghai, China

**Keywords:** breast cancer, gut microbiota, multi-omics integration, serum metabolomics, untargeted metabolomics

## Abstract

**Objective:**

Alterations of the gut microbiota and host metabolic reprogramming are closely associated with the development of breast cancer and the treatment response; however, integrated studies of the gut microbiota and metabolome spanning the transition from benign breast disease (BBD) to malignancy and the postchemotherapy phase remain limited. This study aims to systematically characterize the dynamic changes in the “gut microbiota–serum metabolome–breast tumor” axis from benign breast disease (BBD) to breast cancer (BC) and postchemotherapy breast cancer (PCBC) and to evaluate its potential value in diagnosis and disease monitoring.

**Methods:**

We enrolled 295 female participants, who were divided into a BBD group (n = 83), a BC group (n = 100), and a PCBC group (n = 88), and included 24 paired fecal samples from the same patients that were collected before and after chemotherapy. Fecal samples underwent 16S ribosomal RNA (rRNA) sequencing, while serum samples underwent an liquid chromatography–high-resolution mass spectrometry (LC–MS/MS) based nontargeted metabolomic analysis; we compared differences in gut microbiota diversity, taxonomic composition, and functional predictions across groups and screened for differentially abundant metabolites and enriched metabolic pathways. In a subset of patients with paired multiomics data (BBD n=19, BC n=31, and PCBC n=34), Spearman’s correlation analysis, multiomics principle component analysis (PCA)/partial least squares-discriminant analysis (PLS-DA), and random forest models were employed to integrate the microbiota and metabolic features.

**Results:**

Cross-sectional remodeling of the gut microbiome structure occurred. The α diversity of the gut microbiota was similar across the three groups (BBD, BC, and PCBC); however, the β diversity analysis based on the weighted UniFrac distance revealed the significant separation of the microbial community structure among the three groups. At the taxonomic level, the BBD group was significantly enriched with beneficial commensal bacteria that produce short-chain fatty acids (e.g., *Faecalibacterium* and *Roseburia*); in contrast, the BC group shifted toward the enrichment of inflammation- or tumor-associated genera (e.g., *Blautia*, *Fusobacterium, Sneathia*, and *Prevotella*), while the PCBC group further accumulated various opportunistic pathogens (such as *Phocaeicola, Sutterella, Enterococcus*, and *Chlamydia*). As the disease progressed and chemotherapy was administered, the microbiome gradually shifted from a “metabolic protective” state to an “inflammatory/pathogenic” state. The characteristics of the dynamic remodeling of serum metabolomic profiles were identified. Nontargeted metabolomics revealed more than 3,000 metabolites, and the multivariate analysis indicated significant heterogeneity in the metabolomic profiles associated with malignant transformation and those measured before and after chemotherapy. In the BC group, energy, amino acid, and lipid metabolism were significantly disrupted, and widespread metabolite depletion was observed; however, in the postchemotherapy PCBC group, adaptive pathways such as estrogen, bile acid, and drug metabolism were activated, and persistent abnormalities in purine/nucleotide, carbon, and multiple amino acid metabolism were detected. Overall, the serum metabolic network underwent a dynamic remodeling process, transitioning from homeostasis to severe disruption and then to partial reconstruction following treatment. Among these changes, the differentially abundant metabolites torsemide, cortolone-3-glucuronide, and trimethylselenonium all had area under the curve (AUC) values greater than 0.75 in distinguishing between different disease stages and chemotherapy statuses, demonstrating their good potential as biomarkers. Interaction networks and multiomics predictive models of the “gut–metabolism–tumor” axis were established. Multiomics association networks revealed a systemic shift in gut–metabolism interaction patterns from a steady state characterized by “probiotic–energy/amino acid and polyphenol metabolism coupling” during the benign phase to a new steady state dominated by “proinflammatory/opportunistic pathogens–lipid reprogramming, exogenous metabolite metabolism, and oxidative stress.” A multiomics classification model based on random forests demonstrated that the combined analysis of the gut microbiota and serum metabolite profiles exhibited exceptional efficacy in distinguishing between the BBD, BC, and PCBC groups. It identified a cluster of strongly associated features characterized by anaerobic gram-positive cocci, *Lactobacillus*-associated microbiota, and their paired metabolites, providing an important molecular fingerprint for clinical assessment.

**Conclusions:**

This study integrates gut microbiome and serum metabolomic data to reveal that during the progression of benign breast lesions to breast cancer and throughout chemotherapy, the “gut microbiota–host metabolism–breast tumor” axis transforms from a state characterized by commensal depletion, the expansion of opportunistic pathogens, and reprogramming of energy/lipid metabolism to a persistent metabolic signature associated with drug metabolism and the activation of oxidative stress pathways. The integrated multiomics model helps characterize the biological differences across various stages of breast disease. The identified characteristic bacterial genera and metabolite combinations provide preliminary theoretical clues for the future exploration of microbiome-related mechanisms in breast cancer and for auxiliary assessments.

## Introduction

Breast cancer has become a major public health issue for women worldwide. According to global cancer statistics, its incidence has surpassed that of lung cancer, making it the leading cause of cancer-related deaths among women, accounting for one-sixth of all cancer-related deaths (1). Research into the mechanisms of breast cancer metastasis and its treatment still faces many unresolved challenges. In recent years, a large body of research has demonstrated that the microbiome of the body, particularly the gut microbiota, plays critical roles in tumorigenesis, immune regulation, and the treatment response (2–4).

The gut microbiota is regarded as an “invisible organ”; with a genetic diversity approximately 150 times that of the human genome, it plays crucial roles in maintaining immune balance, nutrient metabolism, and overall health (5–7). In recent years, a growing body of research has revealed the influence of gut microbial diversity on the host immune system, including its roles in tumor initiation, progression, and treatment. Studies have shown that specific microbial communities can influence active estrogen levels in the body by modulating the estrogen cycle (estrobolome) and can participate in the development and progression of breast cancer by regulating estrogen metabolism, inflammatory responses, and immune homeostasis (5–7). Furthermore, metabolites produced by the gut microbiota (such as short-chain fatty acids, bile acids, and tryptophan metabolites) play significant roles in regulating the tumor microenvironment, immune cell differentiation, and chemotherapy sensitivity (8, 9). Molecular subtypes of breast cancer (such as Luminal A, Luminal B, human epidermal growth factor receptor 2 (HER2)-positive, and triple-negative breast cancer (TNBC)) have distinct biological characteristics and clinical behaviors. Studies have shown that patients with different subtypes of breast cancer may exhibit specific differences in the gut microbiota composition (10–12). For example, patients with luminal-type breast cancer tend to have a relatively stable ratio of *Bacillota* to *Bacteroidota*, whereas patients with triple-negative breast cancer exhibit more pronounced microbial alterations and proinflammatory characteristics (13). Although these findings suggest a potential association between the gut microbiota and molecular subtypes of breast cancer, most relevant studies have focused on small samples or single populations, and systematic research—particularly in Asian populations—remains scarce. A systematic analysis of the characteristics of the gut microbiota in patients with breast cancer and their relationships with disease subtypes and the treatment response is highly important for elucidating the microbiological mechanisms underlying this disease. A growing body of evidence suggests that the composition of the gut microbiota is not only closely associated with the development of breast cancer but also may influence its clinical characteristics and progression. In a systematic review of multiple studies, Amaro-da-Cruz et al. (14) noted that the gut microbiota of breast cancer patients exhibits specific imbalances. The abundance of certain genera is significantly associated with patients’ clinical stage, tumor grade, and molecular subtype, suggesting that alterations in the microbiota may contribute to breast cancer progression and increased tumor aggressiveness. As a key component of breast cancer treatment, chemotherapy exhibits significant individual variability in both efficacy and adverse reactions. Recent studies have shown that the gut microbiota can influence the chemotherapy response by modulating drug metabolism, host immune responses, and mucosal barrier function (15–17). Certain beneficial bacteria, such as *Faecalibacterium* and *Bifidobacterium*, can enhance the antitumor effects of immunotherapy and chemotherapy drugs; conversely, the overgrowth of *Bacteroides* and other opportunistic pathogens is closely associated with chemotherapy-related toxicity (such as diarrhea and colitis) (15, 18). However, the systemic effects of chemotherapy on the gut microbiota of breast cancer patients remain unclear, particularly in terms of the differences in the microbiological response to chemotherapy among patients with different molecular subtypes and clinical stages. Furthermore, a complex bidirectional regulatory relationship exists between the gut microbiota and host metabolites (19–21). The gut microbiota influences host energy metabolism, immune responses, and signal transduction processes through its metabolic products. Current research suggests that chemotherapy may lead to significant changes in the levels of short-chain fatty acids and aromatic metabolites and that fluctuations in these metabolites are closely associated with immune activity, the risk of tumor recurrence, and chemotherapy resistance (22, 23). Metabolomics, a key technology for studying host–microbiome interactions, provides a new perspective for elucidating the metabolic links between chemotherapy and changes in the microbiome. The objective of this study was to systematically investigate the differences in the characteristics of the gut microbiota between breast cancer patients and patients with benign breast tumors through gut microbiota profiling and to observe fluctuations in the microbiota composition before and after chemotherapy intervention by comparing the microbiota distribution across different clinical stages and molecular subtypes and analyzing the correlation between chemotherapy-associated microbiota characteristics and host metabolic indicators. We aimed to provide preliminary observational evidence for understanding the clinical features of breast cancer and the systemic response to chemotherapy.

## Materials and Methods

### Participant information

This study was approved by the Ethics Committee of Qinghai University Affiliated Hospital, and all participants signed informed consent forms. The study included a total of 271 female patients with breast diseases confirmed by clinical pathology and imaging, and a total of 295 samples were collected. Participants were divided into three cross-sectional groups based on disease stage: benign breast disease (BBD, n=83), newly diagnosed breast cancer (BC, n=100), and breast cancer after completion of 4 cycles of chemotherapy (PCBC, n=88). Sample allocation for each analysis module is as follows, Fecal microbiome analysis. All 271 baseline fecal samples were included (BBD n=83, BC n=100, PCBC n=88). Serum metabolomics and multi-omics integration analysis: 100 samples with available serum data and acceptable quality were included. To ensure the rigor of cross-omics associations, a total of 84 high-quality samples with paired fecal microbiome and serum untargeted metabolomics data were selected (BBD n=19, BC n=31, PCBC n=34) for multi-omics integration modeling. Longitudinal Chemotherapy Cohort: An additional 24 breast cancer patients provided paired fecal samples before and after chemotherapy (pre-chemotherapy SBC n=24 and post-chemotherapy SPCBC n=24); due to limitations in clinical sampling conditions, corresponding serum samples were not collected for this longitudinal cohort.

The inclusion and exclusion criteria were as follows. Inclusion criteria: (1) participants fully understood the study procedures and voluntarily agreed to participate; (2) patients with breast cancer or suspected benign breast nodules had their diagnoses confirmed by pathological examination; (3) patients in the breast cancer chemotherapy group had not undergone surgical treatment and had completed 4–6 cycles of neoadjuvant chemotherapy; and (4) patients in the breast cancer group had not received chemotherapy, radiotherapy, or other anticancer treatments prior to stool collection. Exclusion criteria for all three groups were: (1) a history of malignancies other than breast tumors, metabolic syndrome, diabetes, autoimmune diseases, or chronic intestinal diseases; (2) use of oral probiotics or antibiotics within the previous 3 months; (3) a history of diarrhea within the previous 3 months; and (4) poor treatment compliance or unwillingness to receive standard medical management.

### Sample collection

Approximately 10 g of fresh morning fecal sample was obtained from each participant, placed in a sterile fecal collection container, and labeled according to the predefined group assignments. The samples were stored at −80 °C within 20 minutes after collection. Venous blood was collected by trained nurses in strict accordance with standard aseptic procedures; serum was separated by centrifugation and stored at −80 °C until analysis.

### Microbial DNA extraction, 16S rRNA gene sequencing, and data processing

Collected samples were preprocessed and subjected to quality assessment before DNA extraction. Fecal DNA was extracted using the MagPure Stool DNA Kit according to the manufacturer’s instructions; after mechanical disruption, lysis, and centrifugation, magnetic bead–based purification was performed on a KingFisher Flex automated platform using the MagPure Stool DNA KF program. The extracted DNA was stored at −25 °C, its concentration and purity were determined using the Qubit dsDNA BR Assay, and its integrity was evaluated by 1% agarose gel electrophoresis. The V3–V4 region of the bacterial 16S rRNA gene was amplified using primers 341F (5’-ACTCCTACGGGAGGCAGCAG-3’) and 806R (5’-GGACTACHVGGGTWTCTAAT-3’), with both forward and reverse primers ligated to Illumina sequencing adapters and linker sequences. For each sample, 30 ng of genomic DNA was used in a 50 μL PCR reaction mixture, and amplification was performed under standard conditions (30 cycles following initial denaturation, with a final extension step). PCR products were purified using Agencourt AMPure XP magnetic beads and used to construct sequencing libraries with adapter and barcode ligation; library quality was assessed with an Agilent 2100 Bioanalyzer, and qualified libraries were sequenced on an Illumina HiSeq platform following standard protocols. Paired-end reads obtained from sequencing were merged based on their overlap using FLASH software (v1.2.11), with a minimum overlap of 15 bp and a maximum mismatch rate of 0.1, to generate hypervariable region (HVR) tag sequences for subsequent analyses.

### LEfSe differential analysis method for gut microbiota

LEfSe (Linear Discriminant Analysis Effect Size) was employed to identify microbial biomarkers with significant abundance differences between groups. This method first uses the non-parametric Kruskal-Wallis (KW) rank-sum test to detect taxonomic units with significant abundance differences across groups, and then utilizes Linear Discriminant Analysis (LDA) to assess the magnitude of the impact of these differential features on the classification performance of each group. When analyzing microbial heterogeneity across different molecular subtypes of breast cancer (Luminal A, Luminal B, HER2+, TNBC), the screening criteria for simultaneous comparison of all four groups were deemed too stringent. Therefore, we adopted a pairwise comparison strategy with greater biological relevance to identify representative differential bacterial genera between subtypes. This strategy aims to capture small but critical subtype-specific deviations in the microbiome, avoiding the excessively high false-negative rate associated with multi-group comparisons. In this study, the significance level was set at *P* < 0.05, and the threshold for the LDA score was set at > 2.0. The analysis results were visualized using a cladogram or a bar chart of LDA score distributions.

### Serum non-targeted metabolomics analysis

Before LC–MS/MS analysis, serum samples were thawed on ice, and an appropriate volume of each was mixed with pre-chilled organic solvent containing internal standards for protein precipitation, followed by vortex mixing, ultrasonication, and centrifugation. The resulting supernatants were used for analysis, and quality control (QC) samples were prepared by pooling equal volumes of supernatant from all samples. Untargeted metabolomic profiling was performed on a high-performance liquid chromatography–high-resolution mass spectrometry (LC–MS/MS) platform, acquiring full-scan data in both positive and negative ion modes. Raw data were processed with dedicated software for peak detection, retention-time correction, peak alignment, and normalization; after removal of noise and features with a high proportion of missing values, metabolites were annotated by matching accurate mass-to-charge ratios and MS/MS fragmentation spectra against the HMDB, METLIN, KEGG and other databases, yielding a quantitative metabolite matrix. Subsequently, the data were imported into statistical software for multivariate analyses, including principal component analysis (PCA) and partial least squares discriminant analysis (PLS-DA), as well as univariate testing. Differential metabolites were screened on the basis of variable importance in projection (VIP) values and p-values, with multiple testing corrected using the false discovery rate (FDR) method. KEGG pathway enrichment analysis was then performed to elucidate the metabolic pathways involved.

### Bioinformatics and statistical analysis

#### 16S rRNA gene sequencing and bioinformatics analysis

FLASH software (v1.2.11) was used to assemble paired-end reads based on overlap (minimum overlap of 15 bp, mismatch rate of 0.1), generating hypervariable region (HVR) tag sequences. Species classification analysis was performed based on the tag sequences, and sequencing depth was assessed using species accumulation curves. α-diversity analysis: ACE, Chao, Shannon, Simpson were calculated to evaluate community richness and evenness. β-diversity analysis: Differences in community structure between groups were assessed using Weighted UniFrac and Unweighted UniFrac distance matrices. Visualization methods include Principal Coordinate Analysis (PCoA), Non-metric Multidimensional Scaling (NMDS), and Venn diagram analysis. Statistical tests employ PERMANOVA (permutation-based multivariate analysis of variance) to assess significance between groups, and PERMDISP (multivariate dispersion homogeneity test) to verify differences in dispersion between groups. Identification of Differential Microbiomes: LEfSe analysis (LDA > 2, *P* < 0.05) was used to identify signature microbial features distinguishing different clinical subgroups. Functional Prediction Analysis: The PICRUSt2 tool was used to predict the inferred functional potential of the gut microbiota in KEGG pathways (secondary and tertiary pathways) based on 16S rRNA data.

### Non-targeted metabolomics analysis

Peak extraction, retention time correction, peak alignment, and normalization were performed using specialized software; metabolite identification was conducted in the HMDB, METLIN, and KEGG databases based on precise mass-to-charge ratios and MS/MS fragmentation patterns. Unsupervised Principal Component Analysis (PCA) is used to assess the overall metabolic distribution; supervised Orthogonal Partial Least Squares Discrimination Analysis (OPLS-DA) is employed to identify key differential variables between groups, with permutation tests used to prevent model overfitting. Screening of differentially expressed metabolites: Differentially expressed metabolites were screened based on VIP values (VIP > 1), P-values (P < 0.05), and fold changes. Metabolic pathway enrichment: Metabolic pathway enrichment analysis was performed based on the KEGG database.

### Multi-omics integration and classification model development

Spearman’s correlation analysis was used to assess the association between gut microbiota and differentially expressed metabolites, and Pearson’s correlation analysis was employed to investigate the correlation between species/metabolites and clinical indicators. Classification Model Development: A Random Forest model was constructed to assess the contribution of microbial and metabolic features to group differentiation, and important features were selected based on the Mean Decrease in Accuracy (MDA) and Mean Decrease in Gini (MDG). The diagnostic performance of candidate microbial and metabolic biomarkers across different disease stages and treatment statuses was evaluated using ROC curves (Receiver Operating Characteristic curves) and their area under the curve (AUC). Adjustment for confounding factors: Multivariate logistic regression models were constructed, incorporating age, menopausal status, and other variables as covariates to account for the potential influence of confounding factors on core microbial and metabolic features.

### Conventional statistical tests

Tests of data distribution: The Shapiro-Wilk test was used to assess normality. Continuous variables: For variables that followed a normal distribution, Student’s t-test or one-way analysis of variance (ANOVA) was used; for those that did not, the Wilcoxon rank-sum test or the Kruskal-Wallis test was used. Categorical variables: Chi-square (χ²) tests or Fisher’s exact tests were used. Multiple comparison correction: P-values were corrected using the FDR (False Discovery Rate) (*P* < 0.05 was considered statistically significant). Software support: All statistical analyses were performed using the R software environment.

## Results

### A cross-sectional comparative study of the gut microbiota in three groups: patients with benign breast tumors, patients with breast cancer, and patients who have undergone chemotherapy for breast cancer

**Figure 1:**
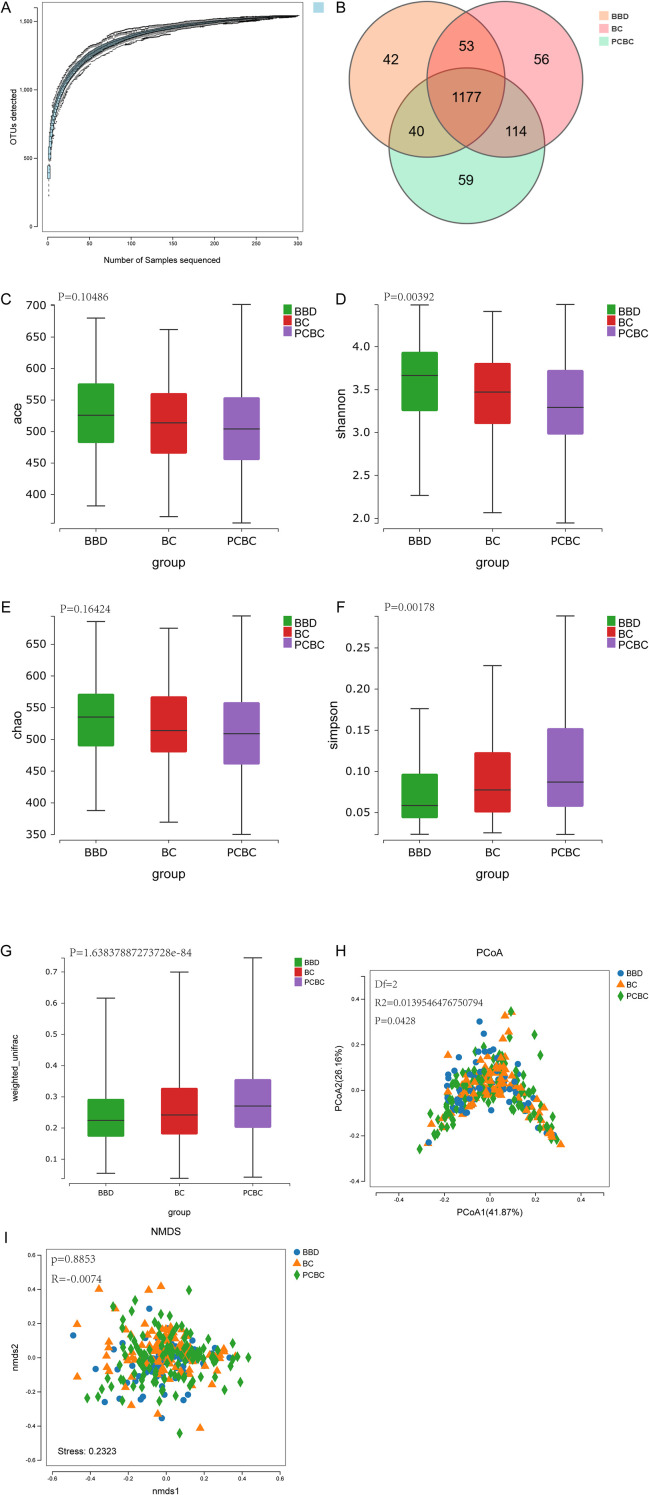
**(A)** The species accumulation curve shows that as the number of randomly selected samples increases, the number of species rises rapidly and then levels off, indicating that the sequencing depth is sufficient to capture the major microbial diversity in the samples. **(B)** The Venn diagram illustrates the distribution of shared and unique genera at the OTU level among three patient groups: benign breast tumors, newly diagnosed breast cancer (BC = 100), and post-chemotherapy breast cancer. **(C, D)** The Shannon and Simpson indices, which reflect species diversity and evenness, were analyzed using the Wilcoxon rank-sum test or the Kruskal–Wallis test; **(E, F)** The Chao and ACE indices, which reflect species richness, were analyzed using the Wilcoxon rank-sum test or the Kruskal–Wallis test. The results showed that the Shannon and Simpson indices differed significantly among groups (P < 0.05), while no statistical differences were observed for the Chao1 and ACE indices. **(G)** β-diversity analysis using PERMANOVA based on Weighted UniFrac distances. Intergroup dispersion was assessed using the PERMDISP test (P < 0.05 was considered significant). **(H)** PCoA analysis based on weighted UniFrac distances. The first two principal coordinates explained 41.87% and 26.16% of the variance, respectively. Samples from different groups showed significant overlap in their spatial distribution, and ANOSIM analysis (R = −0.00745, P = 0.8553) indicated no significant differences in community structure among groups. **(I)** NMDS analysis based on community structure. The stress value (Stress = 0.2369 > 0.2) further indicates that the samples from different groups are poorly distinguished in the low-dimensional space. (BBD = 83), (BC = 100), (PCBC = 88).

This study included patients with benign breast disease (BBD), patients with newly diagnosed breast cancer (BC), and patients with breast cancer who had undergone 4 to 6 cycles of chemotherapy (PCBC). Comparisons of the clinical and pathological characteristics across the groups are shown in **Table 1**. No significant difference in age was observed among the three groups (*P* = 0.967). Since the BBD group consisted of noncancer patients, significant differences in the Ki-67 index and tumor size were observed among the three groups (all *P* < 0.001); however, these differences are clinically reasonable. Further comparisons between the BC group and the PCBC group revealed no significant differences in any clinical or pathological characteristics, including the Ki-67 index, tumor size, T stage, N stage, or molecular subtype (all *P* > 0.05), suggesting that the two groups were comparable at baseline and suitable for the subsequent microbiome analysis. The species accumulation curve confirmed the reliability of the sequencing depth in this study. The Venn diagram shows that at the species composition level, unique microbial species set characteristics were identified between groups with different clinical statuses (**Figures 1A, B**). In terms of α-diversity (**Figures 1C–F**), significant differences were observed in the Shannon and Simpson indices across groups, whereas no significant differences were detected in the Chao1 and ACE indices, which represent species richness. This inconsistency suggests that different disease states and a history of chemotherapy are primarily associated with differences in microbial community evenness rather than overall species richness. This result may imply that, across different clinical contexts, the relative proportions of certain dominant bacterial communities exhibit intergroup heterogeneity, while the total abundance of core species remains relatively constant—a finding consistent with observations from previous studies (24). With respect to β diversity, a permutational multivariate analysis of variance (PERMANOVA) based on weighted UniFrac distances revealed significant differences in community structure among the three groups (*P* < 0.001). Given the sensitivity of PERMANOVA to intergroup dispersion, we supplemented the analysis with a dispersion homogeneity test (permutational multivariate analysis of dispersion, PERMDISP). The results indicated significant heterogeneity in dispersion among the three groups (weighted UniFrac: *F* = 4.40, *P* = 0.013; unweighted UniFrac: *F* = 4.18, *P* = 0.016). Further analysis of the intersample distances within each group revealed that the distribution pattern tended to increase in the order of BBD < BC < PCBC (Kruskal–Wallis *P* < 0.001), and the PCBC group exhibited the greatest intracommunity heterogeneity compared with the BC group (pairwise *P* < 0.001; **Figure 1G**). In summary, the significant differences observed among the BBD, BC, and PCBC groups were driven by a combination of shifts in the community center of mass and changes in intragroup dispersion; in particular, the microbial community in the PCBC group exhibited greater randomness or instability (**Figure 1G**). However, when we used weighted principle coordinate analysis (PCoA) to assess differences in the overall microbial community (PCoA1 = 41.87%, PCoA2 = 26.16%), samples from the BBD, BC, and PCBC groups showed significant overlap in the two-dimensional coordinate space. Further ANOSIM confirmed the lack of statistically significant separation in the abundance-weighted structure of the overall gut microbiota among the three groups (*R* = −0.00745, *P* = 0.8553). The nonmetric multidimensional scaling (NMDS) stress value (stress = 0.2369 > 0.2) also corroborated the weak distinguishability of the samples from each group in the low-dimensional space (**Figures 1H, I**).

**Table 1:** Baseline characteristics of the study population.

Indicators	Category	BBD group(n=83)	BC group(n=100)	PCBC group(n=88)	*χ*²Value¹	*P* Value¹	*χ*²Value²	*P* Value²
Age	≤55 y	52 (62.7%)	63 (63.0%)	54 (61.4%)	0.067	0.967	0.055	0.814
	>55 y	31 (37.3%)	37 (37.0%)	34 (38.6%)				
Menopausal status	Premenopausal	51 (61.4%)	60 (60.0%)	53 (60.2%)	0.052	0.974	0.001	0.977
	Postmenopausal	32 (38.6%)	40 (40.0%)	35 (39.8%)				
T stage	T1	—	28 (28.0%)	35 (39.8%)	—	—	3.372	0.066
	T2-T3	—	72 (72.0%)	53 (60.2%)				
N stage	N-	—	45 (45.0%)	50 (56.8%)	—	—	2.974	0.085
	N+	—	55 (55.0%)	38 (43.2%)				
Clinical stage	I	—	21 (21.0%)	30 (34.1%)	—	—	4.468	0.346
	II	—	61 (61.0%)	44 (50.0%)				
	III	—	15 (15.0%)	12 (13.6%)				
	IV	—	3 (3.0%)	2 (2.3%)				
Molecular subtype	Luminal A	—	23 (23.0%)	17 (19.3%)	—	—	1.392	0.707
	Luminal B	—	45 (45.0%)	42 (47.7%)				
	HER2-enriched	—	13 (13.0%)	16 (18.2%)				
	Triple-negative	—	19 (19.0%)	13 (14.8%)				
Histological grade	Grade1-2	—	78 (78.0%)	60 (68.2%)	—	—	2.608	0.106
	Grade3	—	22 (22.0%)	28 (31.8%)				
Ki-67 index	≤20%	80 (96.4%)	45 (45.0%)	40 (45.5%)	63.482	<0.001	0.005	0.945
	>20%	3 (3.6%)	55 (55.0%)	48 (54.5%)				
Tumor size	≤2 cm	81 (97.6%)	38 (38.0%)	35 (39.8%)	77.294	<0.001	0.072	0.788
	>2 cm	2 (2.4%)	62 (62.0%)	53 (60.2%)				

χ² value¹, P-value¹: Overall comparison of the three groups (BBD group vs. BC group vs. PCBC group) using the chi-square test; applicable only to the three indicators of age, Ki-67 index, and tumor size.χ² value², P-value²: Comparison between the BC group and the PCBC group using the χ² test; applicable to all clinical indicators.“—” indicates that the BBD group is not applicable for this indicator (benign lesions are not included in TNM staging, clinical staging, molecular subtyping, histological grading, or other breast cancer-related staging).Statistical significance is defined as *P* < 0.05.

**Figure 2:**
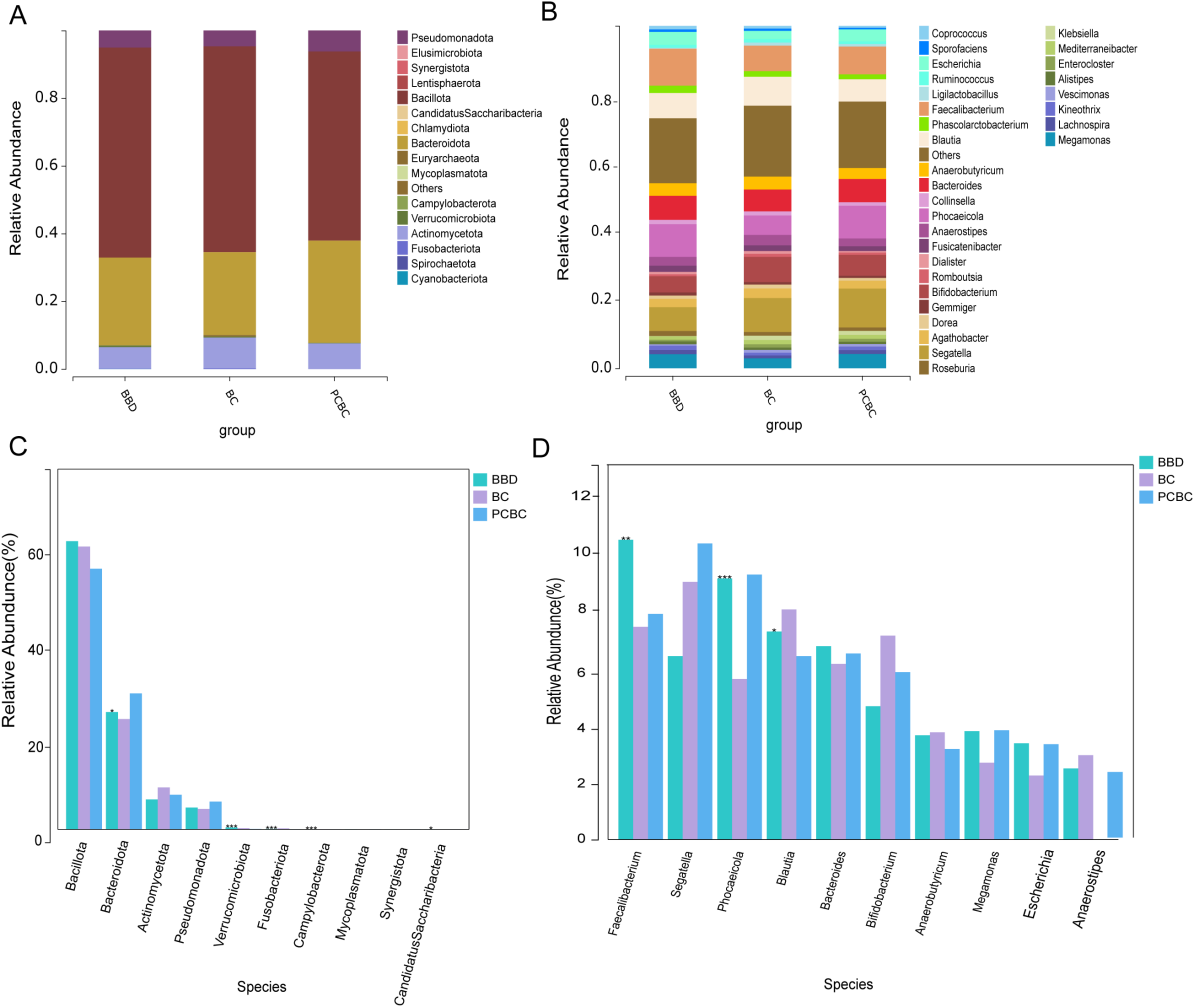
Relative abundance composition of the gut microbiota in the BBD, BC, and PCBC groups at the phylum and genus levels. **(A)** Analysis of species composition at the phylum level for the three groups. **(B)** Analysis of species composition at the genus level for the three groups. **(C)** Stacked bar chart of relative abundance of gut microbiota at the phylum level for the three groups. **(D)** Stacked bar chart of relative abundance of gut microbiota at the genus level across the three groups. (BBD = 83), (BC = 100), (PCBC = 88).

The aforementioned macroecological indicators suggest that despite the differences in the clinical status among the groups, the overall composition and macronetwork structure of their gut microbiota remained relatively stable. Under this relatively stable macrostructure, the microcommunities at specific taxonomic levels exhibited significant intergroup differences. At the phylum level, samples from all three groups shared *Bacillota, Bacteroidota, Actinomycetota*, and *Pseudomonadota* as core dominant groups (total abundance >97%; **Table 2**, **Figure 2A**), further confirming the stability of the core microbiome. Nevertheless, statistically significant differences in the specific composition ratios were observed among the groups (*P* < 0.05). For example, *Bacteroidota* presented the highest relative abundance in the PCBC group (30.16%), which was significantly higher than that in the BBD group (25.92%) and the BC group (24.46%, *P* = 0.0347). Notably, the relative abundances of *Fusobacteriota* and *Campylobacterota*, which were relatively enriched in the BC group, were extremely low in the PCBC group (*P* < 0.001); in contrast, the distribution of *Verrucomicrobiota* decreased across the BBD, BC, and PCBC groups (*P* = 0.0005) (**Figure 2C**). At the genus level, this local remodeling of the microbiome was even more pronounced (**Figures 2B, D**, **Table 3**). The relative abundance of *Faecalibacterium* was highest in the BBD group (10.84%), and was significantly higher than that in the BC and PCBC groups (*P* = 0.0099). Conversely, *Phocaeicola* was present at the lowest abundance in the BC group (5.81%) but was present at higher abundances in the BBD and PCBC groups (*P* = 0.0004). The distribution of *Blautia* also differed significantly among the three groups (*P* = 0.0132). Most other core genera showed no significant differences among the three groups (*P* > 0.05). In addition, certain key butyrate-producing bacteria exhibited different distribution patterns across groups: *Roseburia* was slightly more abundant in the BBD group, while *Klebsiella* was more abundant in the BC and PCBC groups. The phenomenon where certain core short-chain fatty acid-producing bacteria, such as *Faecalibacterium*, exhibit lower abundances in specific groups while other functionally equivalent bacterial groups remain stable suggests the presence of functional redundancy within the gut microbiome network. This redundancy likely maintains the macroscopic community stability observed in the aforementioned NMDS analysis across different clinical states. In summary, specific cross-sectional differences exist in the gut microbial composition among the BBD, BC, and PCBC groups. Although the macrocommunity structure remains relatively stable, at the phylum and genus levels, the relative abundances of groups such as *Bacteroidota, Fusobacteriota, Faecalibacterium*, and *Phocaeicola* significantly differ across the three groups. These distinctive features provide important insights for further exploration of the potential associations between specific gut microbiota and disease states as well as treatment contexts.

**Table 2:** Comparison of relative species abundances (%) among the BBD, BC, and PCBC groups.

Phylum-level species	BBD group (%)	BC group (%)	PCBC group (%)	*P* value
Bacillota	62.04	60.79	55.85	0.0625
Bacteroidota	25.92	24.46	30.16	0.0347
Actinomycetota	6.37	9.09	7.56	0.7299
Pseudomonadota	5.03	4.66	6.19	0.0744
Verrucomicrobiota	0.46	0.27	0.10	0.0005
Fusobacteriota	0.10	0.22	0.03	< 0.001
Campylobacterota	0.006	0.017	0.008	< 0.001
Candidatus_Saccharibacteria	0.004	0.007	0.005	0.0120
Mycoplasmatota	0.012	0.004	0.012	0.6939
Synergistota	0.003	0.007	0.012	0.7856
Others	0.05	0.48	0.05	–
Total	100.00	100.00	100.00	–

ns *p* > 0.05, *p* < 0.05, ** *p* < 0.01, *** *p* < 0.001; relative abundance data are derived from the bar chart data (speciesBar), and p-values are derived from the key species difference table (keySpecies). “Others” includes phyla with lower abundances, such as Cyanobacteriota, Spirochaetota, Euryarchaeota, Chlamydiota, Lentisphaerota, and Elusimicrobiota.

**Table 3:** Comparison of relative abundances of genera among the BBD, BC, and PCBC groups (Top 15, ranked by average abundance).

Genus-level taxa	BBD group (%)	BC group (%)	PCBC group (%)	*P* value
Faecalibacterium	10.84	7.70	8.16	0.0099
Phocaeicola	9.45	5.81	9.59	0.0004
Blautia	7.52	8.33	6.63	0.0132
Bacteroides	6.99	6.35	6.73	0.2189
Segatella	6.64	9.32	10.71	0.2658
Bifidobacterium	4.82	7.37	6.05	0.8038
Megamonas	3.92	2.78	3.95	0.0816
Anaerobutyricum	3.77	3.88	3.27	0.0898
Escherichia	3.48	2.31	3.45	0.9613
Anaerostipes	2.57	3.05	2.30	0.0535
Roseburia	1.48	1.06	1.01	–
Klebsiella	0.12	1.27	1.17	–
Ligilactobacillus	0.27	0.89	0.75	–
Collinsella	1.26	1.20	1.01	–
Ruminococcus	0.87	1.06	0.76	–
Others	35.27	36.78	34.58	–
Total	100.00	100.00	100.00	–

**Figure 3:**
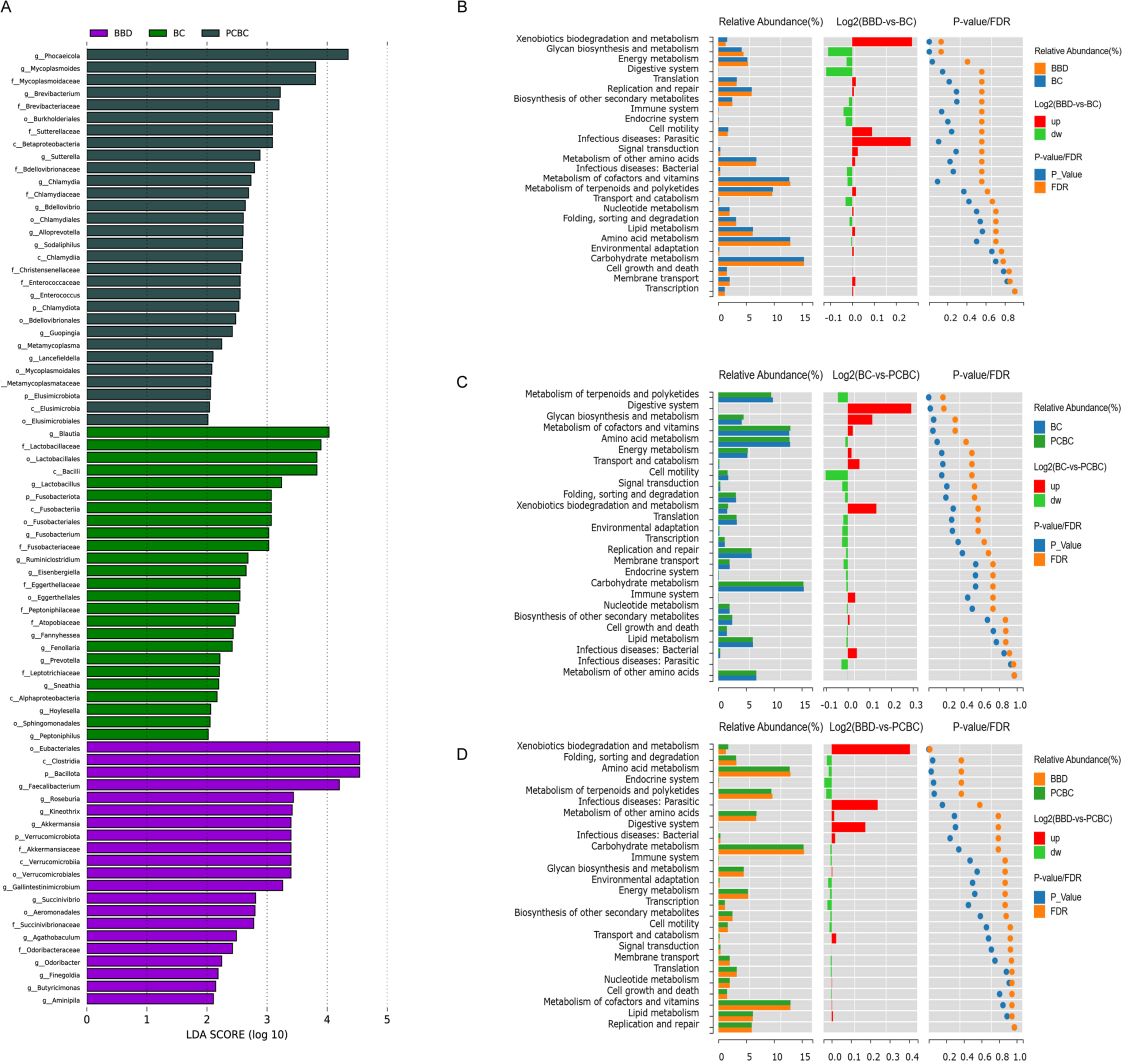
Functional prediction and biomarker analysis of gut microbiota in the BBD, BC, and PCBC groups **(A)** LEfSe analysis of gut microbiota at different disease stages. **(B–D)** Functional differences in gut microbiota based on KEGG Level 2 pathways [**(B)** BBD vs. BC; **(C)** BC vs. PCBC; **(D)** PCBC vs. BBD] (Kruskal–Wallis test, *P* < 0.05). Significantly differentially expressed pathways were identified using LEfSe analysis (LDA > 2, *P* < 0.05). Functional profiles were predicted by the PICRUSt2 software based on the KEGG database.

Linear discriminant analysis effect size (LEfSe) (linear discriminant analysis (LDA) > 2, *P* < 0.05) revealed multilevel microbial signature profiles that differed significantly among the three groups of subjects (**Figure 3A**). The scale of differences across taxonomic levels varied among the groups: the BBD group contained 11 characteristic genera, and the BC group contained 11 characteristic genera, whereas the PCBC group exhibited the broadest taxonomic diversity, involving 12 characteristic genera. The specific distribution of characteristic bacterial communities was as follows: the BBD group was enriched primarily with short-chain fatty acid-producing mucosal commensals such as *Faecalibacterium, Roseburia*, and *Butyricimonas*. The BC group was characterized by the enrichment of inflammation-associated genera such as *Blautia, Lactobacillus, Prevotella*, and *Sneathia.* The PCBC group, in contrast, was characterized by the significant presence of opportunistic pathogens such as *Phocaeicola, Mycoplasmoides, Chlamydia*, and *Enterococcus*. The results of cross-sectional comparisons revealed that the three groups exhibited distinctly different gut microbiome profiles, with differences primarily reflected in the specific enrichment of probiotic-associated bacteria, inflammation-associated bacteria, and opportunistic pathogens across the groups.

We compared the differences in inferred functional potential among the BBD, BC, and PCBC groups of gut microbiota at the Kyoto Encyclopedia of Genes and Genomes (KEGG) level 2 functional level (**Figures 3B–D**). Statistically significant differences were observed across multiple functional categories among the three groups, although the overall magnitude of these differences was small. With respect to the KEGG level 2 pathways, compared with the BBD group, the BC group exhibited a higher predicted relative abundance of “xenobiotic biodegradation and metabolism” but lower predicted relative abundances of “glycan biosynthesis and metabolism” and “energy metabolism” (**Figure 3B**). When the BC and PCBC groups were compared, the predicted relative abundance of “metabolism of terpenoids and polyketides” was slightly lower, while that of “digestive system” was slightly higher; however, the differences in most other pathways were not significant (**Figure 3C**). Compared with the BBD group, the PCBC group had a higher predicted relative abundance of xenobiotic biodegradation and metabolism, while amino acid metabolism and protein folding, sorting, and degradation had slightly lower predicted relative abundances (**Figure 3D**). Statistical tests revealed that the aforementioned differences reached *P* < 0.05 under uncorrected conditions; after the FDR correction, only the difference in xenobiotic biodegradation and metabolism between the PCBC and BBD groups remained significant, suggesting that the predicted functional potential related to xenobiotic processing may be a relatively more stable signal among group differences. The pathway results in this study are derived from functional predictions based on 16S data; the microbial communities may possess different functional potentials rather than directly measured metabolic activities or changes in host physiological functions. At the KEGG level 3 pathway level, the BC group showed predicted enrichment in steroid hormone biosynthesis, glycine/serine/threonine metabolism, riboflavin metabolism, and biotin biosynthesis (all *P* < 0.05). In contrast, the BBD group had higher predicted abundances of toluene degradation, atrazine degradation, and certain pathways associated with pathogen infection (*P* < 0.01) (**Supplementary Figures 1A–C**). Given that this study involved primarily cross-sectional group comparisons and that potential baseline heterogeneity exists among individuals (e.g., diet, lifestyle, age, and comorbidities), the differences in functional predictions are intended mainly to describe group-specific associations and require further validation using metagenomic/metatranscriptomic data and larger-scale longitudinal studies.

### Dynamic changes in the gut microbiota before and after chemotherapy in patients with breast cancer

**Figure 4:**
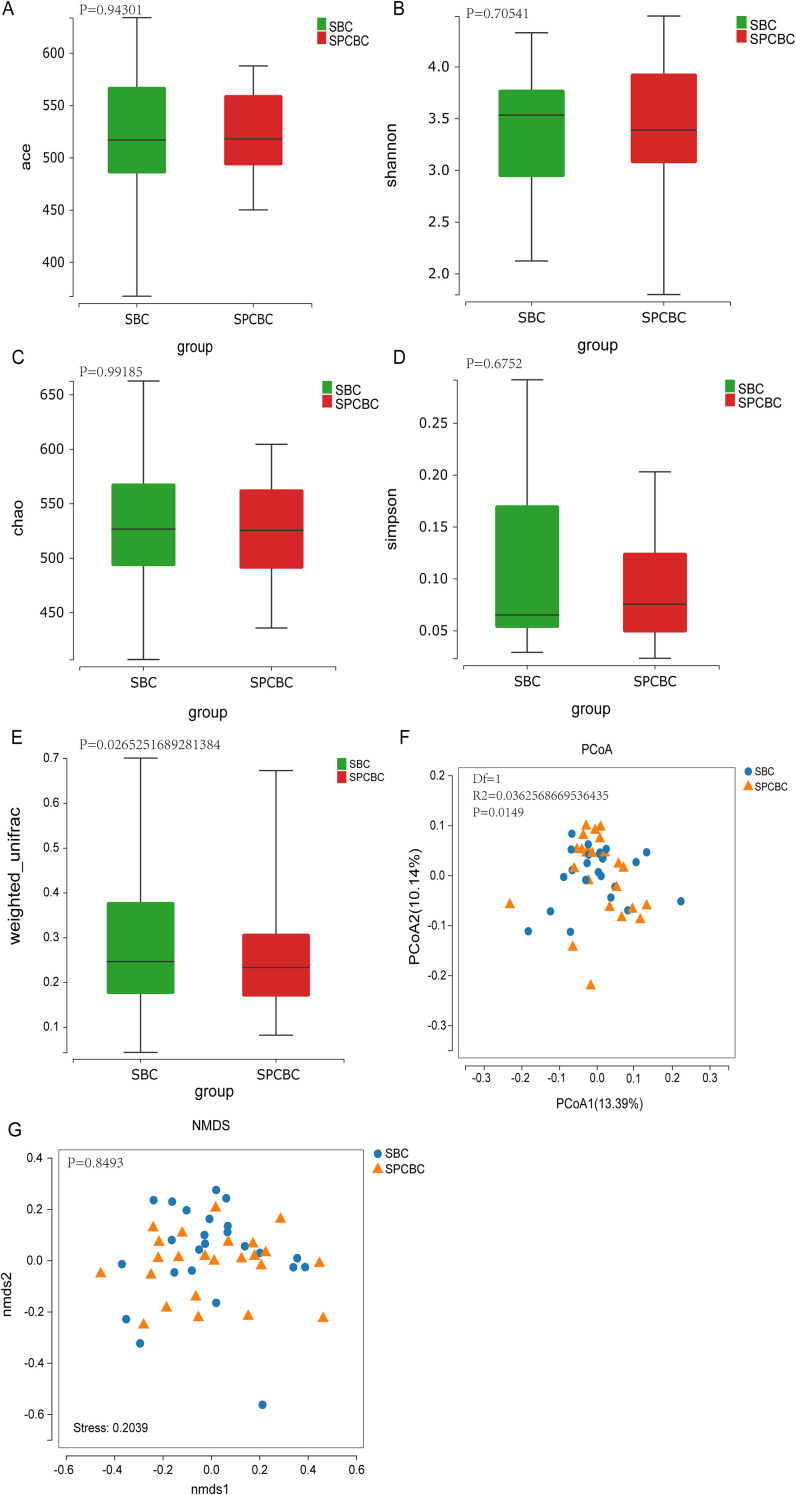
Analysis of α and β diversity in the gut microbiota of breast cancer patients before and after chemotherapy. **(A–D)** Comparison of α-diversity indices (ACE, Chao, Shannon, Simpson) between paired samples before (SBC) and after (PCBC) chemotherapy. Box plots indicate no significant differences in these indices before and after chemotherapy (p > 0.05). **(E)** Comparison of Weighted UniFrac distances, showing a mild but statistically significant reshaping of the microbial community structure after chemotherapy (*P* = 0.0265). **(F)** Principal Coordinate Analysis (PCoA) based on Unweighted UniFrac distances, with explanatory power of 13.86% for PCoA1 and 11.16% for PCoA2. The PERMANOVA test confirmed significant differences in community composition based on species presence/absence *P* = 0.027). **(G)** Non-metric multidimensional scaling (NMDS), Stress = 0.2039; no obvious clustering between groups was observed, and the PERMANOVA test also showed no significant differences (*R* = -0.02475,*P* = 0.8493). (SBC, n=24; PCBC, n=24).

An analysis of paired longitudinal samples (prechemotherapy SBC and postchemotherapy SPCBC) from 24 breast cancer patients revealed that when the pre- and postchemotherapy paired samples were compared, significant differences were not observed in any of the α diversity indices (ACE, Chao, Shannon, and Simpson) before and after chemotherapy (*P* > 0.05), suggesting that chemotherapy did not significantly alter the α diversity of the patients’ gut microbiota (**Figures 4A–D**). PERMANOVA based on the weighted UniFrac distance revealed significant differences in the community structure (*P* = 0.0265). The PERMDISP test further indicated significant differences in dispersion between the pre- and postchemotherapy groups (*F* = 4.2158, *P* = 0.0458). These findings indicate that the effects of chemotherapy not only involved an overall shift in the community composition but also reflected increased heterogeneity within the postchemotherapy group (**Figure 4E**). PCoA based on unweighted UniFrac distances revealed a certain degree of separation in the spatial distribution of the two groups (PCoA1 = 13.86%, PCoA2 = 11.16%). PERMANOVA confirmed that these differences in community composition based on species presence/absence were statistically significant (*P* = 0.027) (**Figure 4F**). However, in the NMDS analysis evaluating the overall community structure, no obvious clustering between groups was observed (stress = 0.2039), and the corresponding statistical test also showed no significant differences between groups (*R* = −0.02475, *P* = 0.8493) (**Figure 4G**). Taken together, these results suggest that chemotherapy-associated changes in the gut microbiota primarily manifest as the appearance or absence of certain species, while the relative abundances of the core microbiota as a whole remain largely stable.

**Figure 5:**
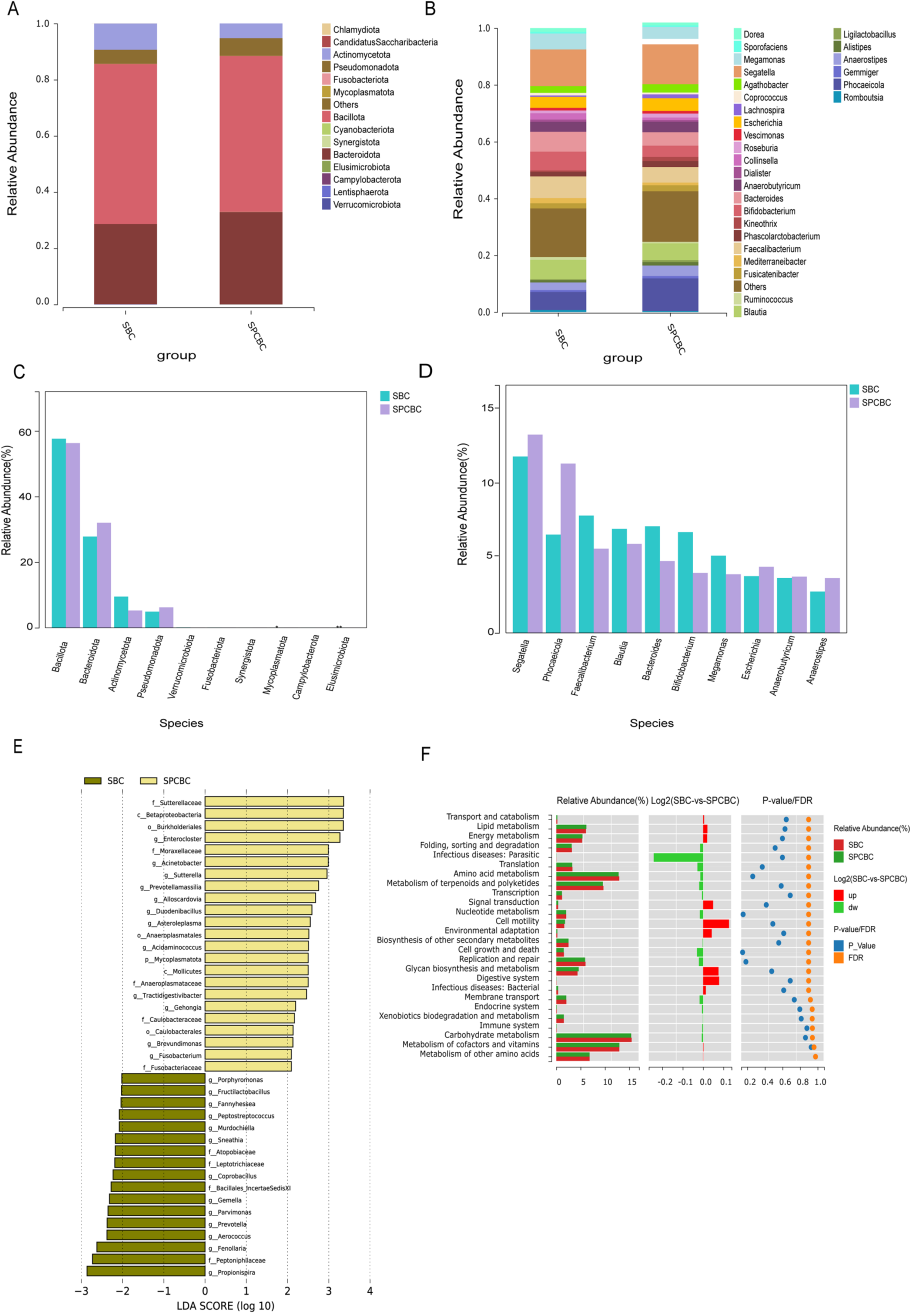
Analysis of gut microbiota species composition, differentially expressed genera, and functional predictions before and after chemotherapy. **(A, C)** Heatmaps and compositional comparisons of relative abundance at the phylum level. **(B, D)** Heatmaps and compositional comparisons of relative abundance at the genus level. **(E)** Significantly different taxonomic units identified by LEfSe analysis (LDA > 2, *P* < 0.05). **(F)** Comparison of relative abundances of inferred KEGG Level 2 functional pathways. Statistical methods: Significantly different pathways were identified using LEfSe analysis (LDA > 2, *P* < 0.05). Functional profiles were predicted by the PICRUSt2 software based on the KEGG database. (SBC, n=24; PCBC, n=24).

Further analysis of species composition confirmed this stability at the macro level. At the phylum level, *Bacillota* and *Bacteroidetes* remained the dominant core components of the microbiota (accounting for >80% of the total). Before and after chemotherapy, the relative abundance of the *Bacillota* phylum decreased only slightly, from 60.3% to 57.5%, while that of the *Bacteroidetes* phylum increased from 26.1% to 29.3%. This fine-tuning of the proportions of these core phyla is highly consistent with the conclusion from the aforementioned NMDS results that significant overall structural bias was not observed (**Figures 5A, C**). However, beneath this relatively stable macrostructure, specific, slight changes occurred at the genus level. The results (**Figures 5B, D**) showed that the relative abundances of typical short-chain fatty acid (especially butyrate) producers, such as *Faecalibacterium* and *Bifidobacterium*, were significantly decreased after chemotherapy, whereas the abundances of the potential opportunistic pathogens *Phocaeicola* and *Escherichia* tended to increase. Notably, the levels of certain key butyrate-producing bacteria, such as *Roseburia* and *Anaerostipes*, remained relatively stable or slightly increased following chemotherapy. This phenomenon suggests the presence of an ecological compensatory mechanism in the gut, whereby the compensatory growth of other butyrate-producing bacteria mitigated the functional deficit caused by the decreased abundance of *Faecalibacterium*, thereby maintaining the overall resilience of the gut microbiome. Overall, in this longitudinal paired cohort, chemotherapy did not lead to a catastrophic disruption of the gut microbiota but rather triggered adjustments in the relative abundances of key bacterial genera and compensatory remodeling of the microbial network.

LEfSe of differentially expressed microbiota (LDA > 2, *P* < 0.05) based on genus-level abundance revealed differences in the fecal microbiota between the SBC and SPCBC samples from the same patient, revealing a total of 30 significantly differentially expressed taxonomic units, of which 11 were enriched in the SBC group and 19 were enriched in the SPCBC group (**Figure 5E**). The SBC group was enriched primarily with *Sneathia*, *Luoshenia*, *Prevotella*, *Metaprevotella*, *Morganella*, *Propionispira*, *Fenollaria*, *Peptoniphilaceae*, and *Clostridium sensu stricto*. The SPCBC group was enriched primarily with taxonomic units such as *Enterocloster*, *Sutterellaceae*, *Betaproteobacteria/Burkholderiales*, *Moraxellaceae/Acinetobacter*, *Pseudomonadales, Duodenibacillus*, *Acidaminococcus*, *Prevotellamassilia*, *Fusobacterium/Fusobacteriaceae*, and *Mycoplasmatota–Mollicutes–Anaeroplasmataceae*. Overall, the paired longitudinal results indicated significant differences in the composition of signature microbial communities before and after chemotherapy, and the taxonomic units that were enriched after chemotherapy included various opportunistic pathogens and gram-negative bacteria.

In terms of predicted functions, the KEGG level 2 functional profiles included primarily amino acid metabolism, carbohydrate metabolism, energy metabolism, membrane transport, and genetic information processing (**Figure 5F**). Compared with the SBC samples, the SPCBC samples presented higher predicted relative abundances of pathways related to membrane transport, cell motility, and signal transduction, whereas pathways related to carbohydrate metabolism, lipid metabolism, and energy metabolism presented lower predicted relative abundances (*P* < 0.05). In addition, pathways related to environmental adaptation and xenobiotic biodegradation and metabolism had higher predicted relative abundances after chemotherapy; conversely, pathways related to fatty acid metabolism and amino acid biosynthesis associated with symbiotic metabolism had lower predicted relative abundances. Notably, the above results, such as those for “xenobiotic metabolism,” reflect differences in predicted functional potential. Approximately 250 pathways were predicted at the KEGG level 3 pathway level. The differential abundance analysis revealed that pathways such as glutathione metabolism, lipopolysaccharide biosynthesis, ABC transporters, and drug metabolism-cytochrome P450 exhibited higher predicted relative abundances in postchemotherapy samples (**Supplementary Figure 2**). Among these pathways, pathways related to steroid hormone biosynthesis and cytochrome P450 should also be interpreted as having inferred functional potential and do not represent direct changes in actual metabolic flux or host drug metabolic capacity. Conversely, pathways such as butanoate metabolism, propanoate metabolism, the TCA cycle, and several amino acid biosynthesis-related pathways exhibited lower inferred relative abundances. Additionally, pathways such as fatty acid β-oxidation, purine metabolism, and polyamine biosynthesis exhibited higher predicted relative abundances in postchemotherapy samples. Collectively, these results suggest differences in the functional potential of the microbiota before and after chemotherapy; however, further validation via metagenomic/transcriptomic or metabolomic analyses is needed.

### Associations between the clinicopathological characteristics of breast cancer and the gut microbiota

**Figure 6:**
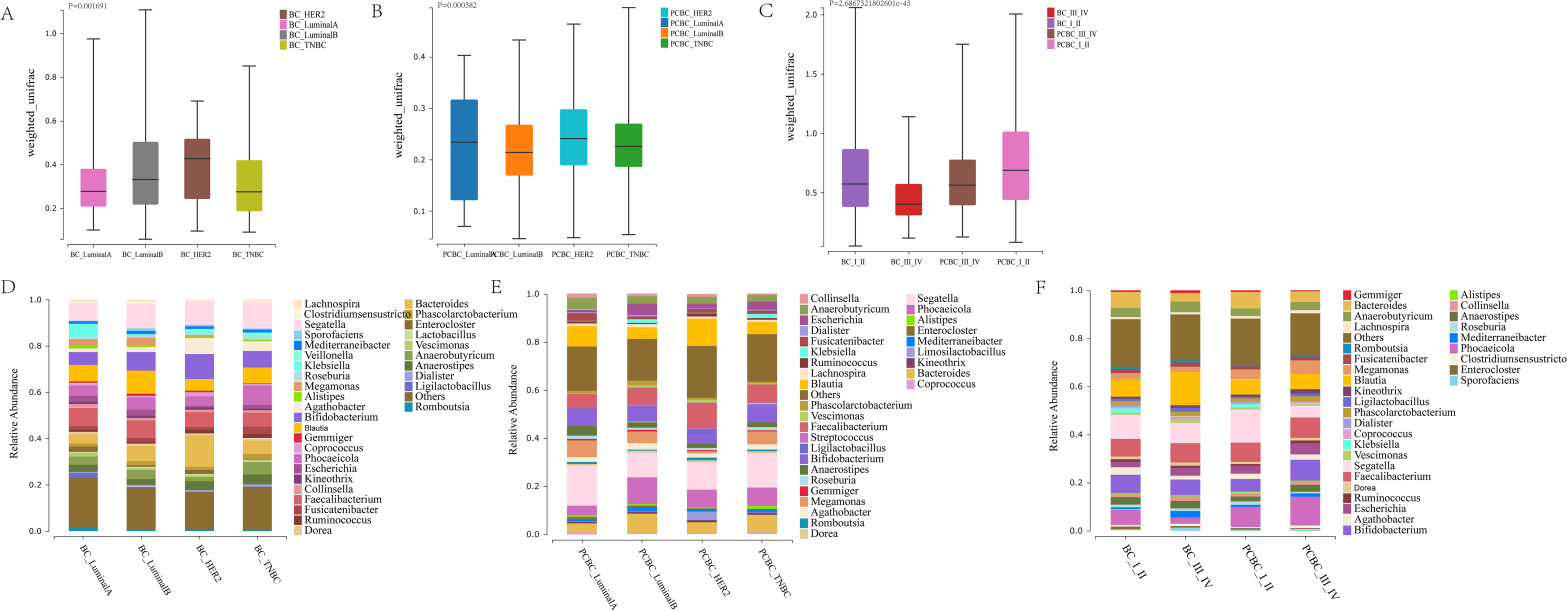
Characteristics of gut microbiota composition in breast cancer patients with different molecular subtypes and clinical stages before and after chemotherapy. **(A, B)** β-diversity analysis based on UniFrac distance between the BC group and the PCBC group showed significant differences in the BC group (**A**, *P* = 0.0017) and the PCBC group (**B**, *P* = 3.82 × 10^-4^). **(C)** Comparison of gut microbiota β-diversity differences between the BC and PCBC groups across different clinical stages (Stages I–II vs. Stages III–IV) (C, *P* = 2.69 × 10^-43^). **(D)** Genus-level composition of the gut microbiota in patients with different molecular subtypes in the BC group. **(E)** Distribution of gut microbiota at the genus level among breast cancer patients with different molecular subtypes in the PCBC group. **(F)** Distribution of gut microbiota at the genus level among breast cancer patients in the BC and PCBC groups based on clinical stage.(BC-LuminalA=13,BC-LuminalB=32,BC-HER2 = 6, BC-TNBC=11;PCBC-Luminal A = 9,PCBC-Luminal B = 44,PCBC-HER2 = 14, PCBC-TNBC = 19;BC-I-II = 56, BC-III-IV = 11, PCBC-I-II = 58, PCBC-III-IV = 16).

Significant association between breast cancer molecular subtypes and clinical stages with gut microbiota α diversity (as measured by the ACE, Chao, Shannon, and Simpson indices) were not observed. In both the BC and PCBC groups, microbial richness and diversity remained stable across different clinical and pathological subgroups (**Supplementary Figure 3**). An analysis of β diversity based on the UniFrac distance was performed to assess the associations between the gut microbiota and molecular subtypes of breast cancer and clinical stages. The results showed significant differences in the gut microbiota structure among patients with the four molecular subtypes (Luminal A, Luminal B, HER2^+^, and TNBC) in the BC group (*P* = 0.0017) (**Figure 6A**). The differences in the microbiome among the patients with different molecular subtypes in the PCBC group remained significant (*P* = 3.82 × 10^-4^), suggesting that some molecular subtype-associated microbiome characteristics may persist after chemotherapy (**Figure 6B**). For further analysis, patients were grouped into four categories based on the clinical stage and chemotherapy status (BC I–II, BC III–IV, PCBC I–II, and PCBC III–IV) for comparison. The results revealed extremely significant differences in the gut microbiota structure across these groups (*P* = 2.69 × 10^-43^) (**Figure 6C**).

An analysis of the genus-level species composition based on different molecular phylotypes (**Figures 6D, E**) revealed that the overall dominant phyla in the BC and PCBC groups remained relatively stable, with both groups being dominated by *Bacillota* and *Bacteroidota*. Moreover, core genera such as *Faecalibacterium*, *Segatella*, and *Blautia* were universally present across patients with different molecular subtypes, clinical stages, and chemotherapy statuses in cross-sectional comparisons, forming a relatively stable framework of the core gut microbiota. A further stratified analysis revealed specific compositional differences among molecular subtypes. In the BC group (**Figure 6D**, **Table 4**), the relative abundance of *Segatella* was higher in patients with subtypes characterized by greater invasiveness (such as HER2-positive and TNBC); conversely, *Faecalibacterium*, which is presumed to have the potential to produce short-chain fatty acids, was more abundant in patients with the Luminal A subtype but relatively lower in patients with TNBC. These findings suggest a cross-sectional association between highly aggressive breast cancer subtypes and a reduction in the abundance of potentially beneficial species in the gut microbiota, as well as an increase in inflammation-associated taxonomic units. In the PCBC group (**Figure 6E**, **Table 5**), the microbial differences between patients with different molecular subtypes tended to homogenize to some extent, yet retained partial subtype-specific signatures. For example, *Faecalibacterium* maintained a relatively high abundance in HER2-positive patients. In Luminal B and TNBC patients, the relative abundance of the potential opportunistic pathogen *Klebsiella* was observed to increase after chemotherapy (Luminal B: 0.30% to 1.52%; TNBC: 0.04% to 1.72%). Whether this reflects a consistent shift in gut microbiota structure following chemotherapy may warrant further investigation.

**Table 4:** Trends of key genera across different molecular subtypes in the breast cancer group.

Category	Genus	Luminal A	Luminal B	HER2+	TNBC	Trend and interpretation
Potentially pathogenic	Segatella	7.45%	10.72%	10.24%	11.38%	Increases with the rising aggressiveness of molecular subtypes
	Klebsiella	5.34%	0.30%	0.24%	0.04%	Shows a burst-like expansion specifically in the Luminal A subtype
	Veillonella	0.24%	0.28%	1.54%	1.75%	Becomes more abundant in the HER2+ and TNBC subtypes
Beneficial genera	Faecalibacterium	8.05%	7.66%	6.37%	5.93%	Decreases progressively with increasing aggressiveness of molecular subtypes
	Anaerobutyricum	3.48%	4.05%	1.85%	5.23%	Shows the highest abundance in the TNBC subtype
	Fusicatenibacter	1.53%	1.54%	1.05%	3.09%	Shows the highest abundance in the TNBC subtype
Fluctuating genera	Bacteroides	3.93%	6.53%	13.57%	5.69%	Is abnormally enriched in the HER2+ subtype
	Bifidobacterium	5.48%	8.08%	10.99%	7.02%	Shows the highest abundance in the HER2+ subtype
	Phocaeicola	4.46%	5.23%	4.14%	8.40%	Shows the highest abundance in the TNBC subtype

**Table 5:** Post-chemotherapy distribution and variation patterns of key genera across molecular subtypes.

Category	Genus	Luminal A	Luminal B	HER2+	TNBC	Variation characteristics
Core genera	*Segatella*	16.58%	10.19%	11.36%	14.29%	Remains at a high level across all subtypes
	*Faecalibacterium*	5.68%	7.18%	10.57%	7.52%	Shows the best recovery in the HER2+ subtype
	*Phocaeicola*	4.10%	11.15%	7.30%	7.60%	Is abnormally enriched in the Luminal B subtype
Opportunistic pathogens	*Klebsiella*	0.15%	1.52%	0.23%	1.72%	Indicates the highest risk in the TNBC subtype
	*Escherichia*	1.10%	4.65%	1.93%	3.31%	Is significantly increased in the Luminal B subtype
Other characteristic genera	*Megamonas*	6.89%	4.80%	0.92%	5.39%	Shows a marked predominance in the Luminal A subtype

Cross-sectional comparisons adjusted for the clinical stage also revealed differences in microbial composition (**Figure 6F**, **Table 6**). In the BC group, compared with patients with early-stage (stage I–II) tumors, patients with late-stage (stage III–IV) tumors had lower relative abundances of *Phocaeicola* in the gut, while the abundances of genera presumed to be associated with inflammation, such as *Blautia* and *Clostridium sensu stricto*, were higher, suggesting that advanced breast cancer may be associated with more significant gut community reorganization. In the PCBC group, the relative abundance of *Phocaeicola* was higher in both patients with early-stage and advanced-stage disease than in the BC group, whereas the relative abundance of potential opportunistic pathogens such as *Enterocloster* was relatively lower. These differences suggest that exogenous exposure to chemotherapy may be associated with alterations in specific gut microbial niches, although this characteristic was not fully evident in the advanced-stage patient group. In summary, cross-sectional data from breast cancer patients with different molecular subtypes and clinical stages revealed characteristic gut microbiota compositions. Chemotherapy intervention is associated with partial structural shifts in the gut microbiome, but these shifts do not fully mask the inherent heterogeneity of tumor subtypes and stages. Furthermore, a multivariate binary logistic regression model adjusted for age and menopausal status as covariates was constructed in this study to further ensure the reliability of the aforementioned clinical observations and to rule out the influence of potential confounding factors (**Supplementary Table 1**). After adjusting for the aforementioned confounding factors, the core differentially abundant bacterial genera identified in this study (such as *Faecalibacterium* and *Blautia*) continued to show significant independent predictive value (independent *P* < 0.05). This result provides strong statistical evidence that the restructuring of the gut microbiota observed in this study is primarily driven independently by disease progression and chemotherapy intervention rather than being confounded by baseline demographic differences among patients.

**Table 6:** Trends of key genera across different clinical stages before and after chemotherapy in breast cancer.

Key genera	BC I–II (early stage, pre-chemotherapy)	BC III–IV (advanced stage, pre-hemotherapy)	PCBC I–II (early stage, post-chemotherapy)	PCBC III–IV (advanced stage, post-chemotherapy)
Blautia	7.3%	14.0%	6.1%	6.5%
Segatella	10.1%	8.4%	13.8%	4.9%
Phocaeicola	6.3%	2.6%	8.5%	12.0%
Bifidobacterium	7.5%	6.5%	5.4%	8.7%
Escherichia	2.2%	3.5%	3.3%	4.6%
Megamonas	2.6%	2.2%	4.3%	5.6%
Faecalibacterium	7.4%	8.0%	7.9%	8.4%

**Figure 7:**
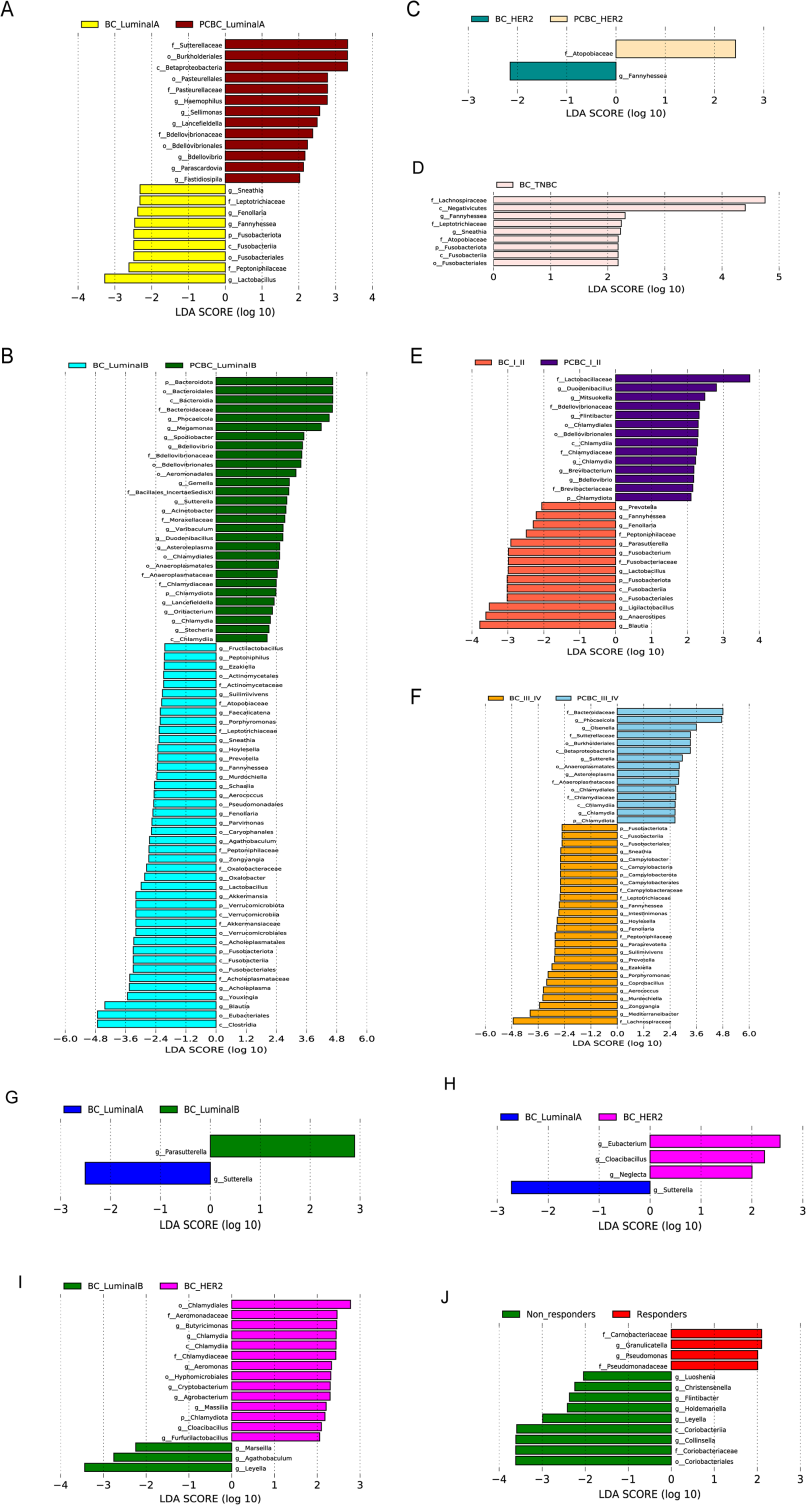
Differential gut microbiota features based on LEfSe analysis (LDA > 2, P < 0.05). **(A–D)** Comparison of microbiota differences between the BC group and the PCBC group stratified by molecular subtype. **(A)** BC_Luminal A vs. PCBC_Luminal B. **(B)** BC_Luminal B vs. PCBC_Luminal B. **(C)** BC_HER2^+^ vs. PCBC_HER2^+^. **(D)** BC_TNBC vs. PCBC_TNBC. **(E, F)** Comparisons stratified by clinical stage. **(E)** BC_I–II vs. PCBC_I–II. **(F)** BC_III–IV vs. PCBC. **(G–I)** Comparison of microbiomes among different molecular subtypes within the BC group. **(G)** Luminal A vs. Luminal B. **(H)** Luminal A vs. HER2^+^. **(I)** Luminal B vs. HER2^+^. **(J)** Stratified analysis within the PCBC group based on response to neoadjuvant chemotherapy.

We compared the LEfSe results between the BC group and the PCBC group at the genus level to systematically evaluate the characteristics of microbiome heterogeneity associated with the chemotherapy status of patients with different molecular subtypes and clinical stages of breast cancer. The results showed that differences in the gut microbiota composition between the two groups exhibited significant subtype and stage dependence. In cross-sectional comparisons across different molecular subtypes, patients with the Luminal A subtype in the BC group primarily were enriched with anaerobic bacteria associated with the *Bacillota* and *Actinobacteria* phyla, such as *Coriobacteriaceae* and *Atopobiaceae*, suggesting that they may possess specific microenvironmental characteristics; in contrast, the PCBC group exhibited a significantly higher relative abundance of *Gammaproteobacteria* within the *Pseudobacteria* phylum, indicating a compositional shift from strict anaerobes to facultative anaerobes across groups (**Figure 7A**). Patients with the Luminal B subtype in the BC group exhibited a dominance of *Clostridia* and *Eubacteriales* within the *Bacillota* and *Bacteroidota* phyla, suggesting that their microbiota maintains a certain potential for fermentative metabolism, whereas in the PCBC group, *Bacteroidia* and *Chlamydiota* were significantly enriched, suggesting that chemotherapy exposure may be associated with an increase in the abundance of certain gram-negative aerobic bacterial groups across groups (**Figure 7B**). Notably, in both groups, HER2-positive patients exhibited *Actinomycetota* and its subordinate taxa *Coriobacteriia* and *Atopobiaceae* as core taxonomic units, with higher LDA values observed in the PCBC group, suggesting that these microbial communities exhibit strong intergroup stability in this subtype (**Figure 7C**). In TNBC patients in the BC group, *Fusobacteriota*, *Actinomycetota*, and *Negativicutes*—taxonomic units presumed to be associated with inflammation—were enriched, while the relative abundances of these microbial communities were lower in TNBC patients in the PCBC group, suggesting that specific microbiome characteristics are differentially associated with the chemotherapy status of patients with this subtype (**Figure 7D**). A comparison of clinical stages further revealed that in patients with advanced-stage (stage III–IV) tumors (**Figure 7F**), *Clostridia* and *Bacteroidia* were enriched in the BC group and primarily exhibited a predicted potential for short-chain fatty acid synthesis and carbohydrate fermentation, whereas in the PCBC group, the dominant phyla were primarily *Chlamydiota* and *Pseudomonadota* (e.g., *Chlamydia* and *Betaproteobacteria*), suggesting that chemotherapy exposure in patients with advanced-stage tumors may be associated with a higher proportion of intestinal facultative anaerobes and potential oxidative stress. In contrast, microbial differences between the two groups were relatively mild in patients with early-stage (stage I–II) tumors, manifesting only as slightly lower *Bacillota* abundance and a slight increase in *Bacteroidota* abundance in the PCBC group, suggesting that the gut microbiome structure of early-stage patients exhibits greater intergroup similarity under different intervention conditions (**Figure 7E**). Overall, LEfSe-based cross-group comparisons indicate that differences in the gut microbiota between the BC and PCBC groups exhibit significant phenotypic and staging heterogeneity, particularly in patients with advanced and highly aggressive subtypes, and are characterized by lower abundances of anaerobic fermenters and relatively higher abundances of facultative anaerobes and aerobic bacteria in the PCBC group.

We clarified the relationships between the gut microbiota and breast cancer molecular subtypes as well as chemotherapy efficacy by restricting the LEfSe to the BC group (breast cancer patients) to compare differences in the gut microbiota between patients with the Luminal A, Luminal B, and HER2+ subtypes (**Figures 7G–I**). Additionally, for PCBC patients who received neoadjuvant chemotherapy, we performed a stratified analysis based on the chemotherapy response (responders vs. nonresponders) (LDA > 2, *P* < 0.05). In the comparison of molecular subtypes within the BC group, a comparison between the Luminal A and Luminal B groups revealed that the Luminal B group was enriched in the genus *Parasutterella*, while the Luminal A group was enriched in the genus *Sutterella*. A comparison between the Luminal A and HER2+ groups showed that the Luminal A group was enriched in the genus *Sutterella*, whereas the HER2+ group was enriched in the genera *Eubacterium*, *Cloacibacillus*, and *Neglecta*. A comparison between Luminal B and HER2+ groups revealed that the Luminal B group was enriched in the genera *Leyella*, *Agathobaculum*, and *Marseilla*; the HER2+ group was enriched in 14 differentially expressed species, primarily the genera *Chlamydia*, *Aeromonas*, *Butyricimonas*, and *Agrobacterium*. In the analysis of PCBC patients stratified by the response to neoadjuvant chemotherapy (**Figure 7J**), the nonresponder group was enriched with 9 differentially abundant species, primarily from the class *Coriobacteriia* and its subordinate taxonomic units (order *Coriobacteriales*, family *Coriobacteriaceae*, genus *Collinsella*), the family *Christensenellaceae* (genera *Christensenella* and *Luoshenia*), *Holdemanella*, *Leyella*, and *Flintibacter*. The responder group was enriched with 4 differentially expressed species, primarily from the genus *Pseudomonas* and related taxonomic units (family *Pseudomonadaceae*) and the genus *Granulicatella* and related taxonomic units (family *Carnobacteriaceae*).

### Analysis of serum metabolic profiles associated with breast cancer development and chemotherapy

**Figure 8:**
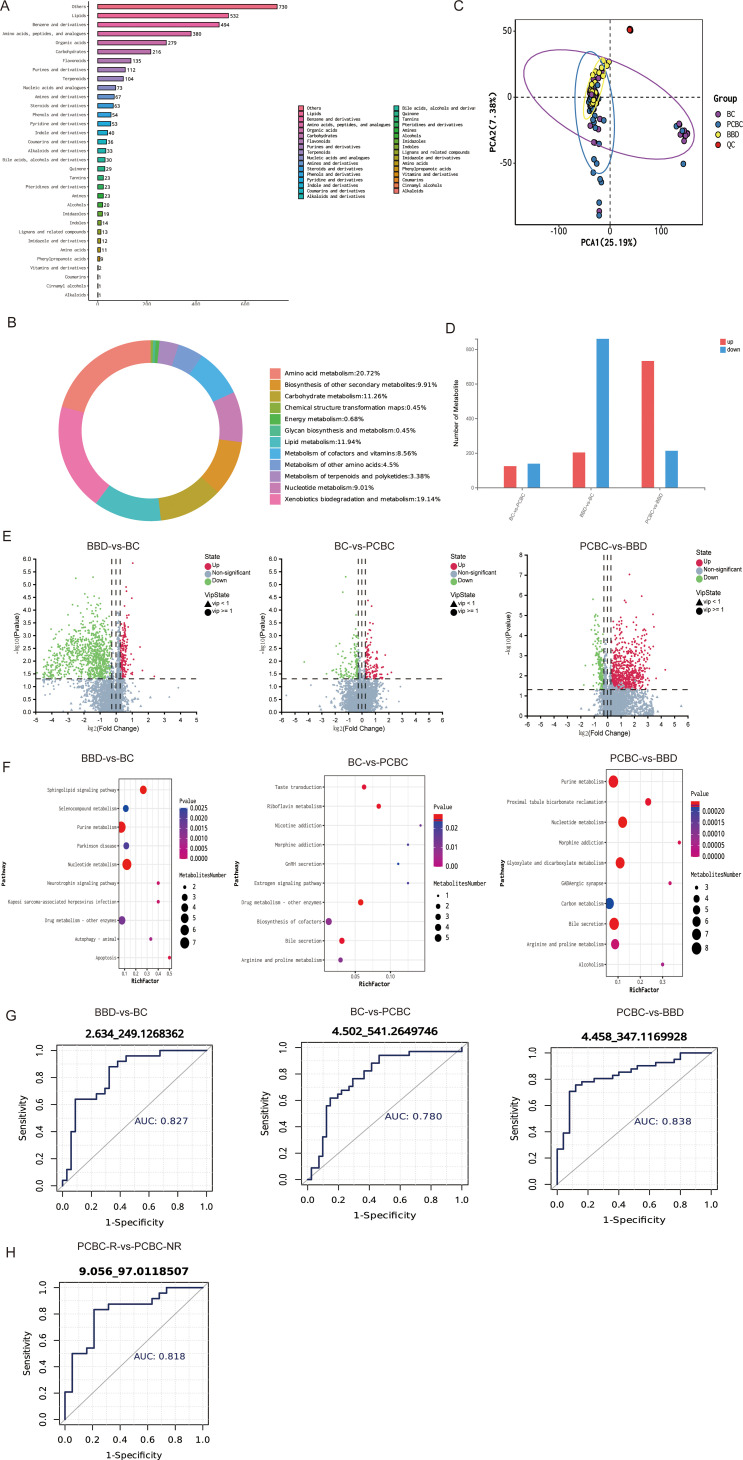
Overall analysis of serum non-targeted metabolomics and screening for differentially expressed metabolites. **(A, B)** Distribution of identified metabolites by chemical structure and biological function. **(C)** Principal component analysis (PCA) score plots of serum metabolomic profiles for the BBD, BC, and PCBC groups. QC samples are highly clustered, confirming the stability of the analytical system. The PERMANOVA test revealed highly significant differences in metabolic profiles among the three groups (F = 33.66, P = 0.001). **(D–F)** Volcano plots of pairwise comparisons: BBD vs. BC **(D)**, BC vs. PCBC **(E)**, and PCBC vs. BBD **(F)** (Wilcoxon test, *q* < 0.05, |log_2_FC| > 0.585). **(F)** ROC curve analysis of representative differentially expressed metabolites across different clinical statuses (BBD vs. BC, BC vs. PCBC, PCBC vs. BBD) (AUC > 0.75). **(G)** Analysis of the inferred functional potential of microbial communities across groups based on the KEGG database. **(H)** (R/NR-related) ROC curve analysis of the metabolite fluoroacetaldehyde between chemotherapy responders and non-responders in the PCBC group (AUC = 0.818). (BBD = 19, BC = 31, PCBC = 34).

An untargeted metabolomic analysis of serum identified more than 3,000 metabolites. The metabolites were classified by their chemical structures and biological functions (**Figure 8A**), and lipids and their derivatives accounted for the highest proportion, followed by benzene ring compounds, amino acids and their analogs, organic acids, and carbohydrates. The analysis also included various secondary metabolites, such as flavonoids, purines/pyrimidines, and terpenoids. KEGG functional annotations (**Figure 8B**) revealed that these metabolites were enriched primarily in pathways related to amino acid metabolism, lipid metabolism, carbohydrate metabolism, and xenobiotic degradation, suggesting that metabolic differences among clinical subgroups were concentrated mainly in networks associated with energy metabolism, substance synthesis, and xenobiotic processing. First, unsupervised PCA was employed in this study to evaluate the overall metabolic distribution characteristics among the three sample groups: BBD, BC, and PCBC. The results showed that the first two principal components (PC1 and PC2) explained 25.19% and 7.38% of the total metabolic variance, respectively (**Figure 8C**). In the PCA score plot, quality control (QC) samples were highly clustered, confirming the stability of the analytical system and the reliability of the experimental data. In terms of sample clustering trends, samples from the BBD group exhibited a relatively compact distribution, reflecting high consistency in the metabolic profiles within the group; in contrast, the samples from the BC group were more widely dispersed and clearly deviated from the samples from the BBD group, suggesting significant individual metabolic heterogeneity within the population of patients with newly diagnosed breast cancer. The distribution pattern of the PCBC group fell between that of the BBD and BC groups, with partial overlap with the BC group. PERMANOVA was conducted to further validate the observed intergroup differences. The results indicated extremely significant differences in metabolic profiles among the three groups (*F* = 33.66, *P* = 0.001), statistically confirming that the pathological progression of breast cancer significantly disrupted serum metabolic homeostasis in the body. Subsequently, we attempted to construct a supervised OPLS-DA model to identify key differential variables. Although the permutation test indicated that the model did not exhibit overfitting (all Q2 intercepts were less than 0), due to the high metabolic heterogeneity within the BC group, the predictive ability (Q2) of the pairwise comparison model did not reach an ideal level.

Pairwise comparisons were conducted among the BBD, BC, and PCBC groups to ensure the rigor of the differentially abundant metabolite screen and the robustness of the results and to systematically elucidate the dynamic changes in serum metabolic profiles during the progression from benign breast lesions to breast cancer and during chemotherapy intervention. Differentially expressed metabolites were identified using the Wilcoxon rank-sum test combined with fold change (*q* < 0.05, |log_2_FC| > 0.585), and the results are presented in volcano plots (**Figures 8D–F**). The analysis revealed that the number of differentially expressed metabolites gradually increased with disease progression and chemotherapy intervention, suggesting a progressive intensification of metabolic reprogramming. A total of 1,097 differentially expressed metabolites were identified in the comparison between the BBD and BC groups. Under the |log_2_FC| ≥ 1 criterion, the levels of only 7 metabolites were significantly increased in the BBD groups, including endogenous steroid hormone metabolites such as 5α-pregnan-3β and 20β-diol-3-sulfate, as well as exogenous drug signals such as tramadol, reflecting symptomatic analgesic treatment for benign lesions. In contrast, the levels of 631 metabolites were significantly increased in the BC group. We clearly classified these compounds into endogenous metabolites and exogenous exposure markers based on their biological origins. Lipid metabolites, such as LysoPC (0:0/18:2(9Z,12Z)), were significantly enriched, objectively reflecting the extensive and profound endogenous metabolic reprogramming that occurs during tumorigenesis; additionally, significant differences in signals associated with drugs and exogenous substances, such as mitozolomide, were also observed in the BC group. This result suggests that the process of malignant transformation from benign breast lesions is not only accompanied by the remodeling of the host’s endogenous metabolic network but also involves intergroup heterogeneity in the exposome. A total of 318 differentially expressed metabolites were identified in the comparison between the BC and PCBC groups. Among these metabolites, differences in endogenous metabolites were primarily concentrated in the remodeling of lipid metabolic networks (such as the dynamic decrease in specific hemolytic phosphatidylcholine), reflecting the partial correction of tumor-associated endogenous metabolic abnormalities by chemotherapy; however, since patients in the PCBC group underwent intensive medical interventions, the majority of metabolites with high discriminative power (high variable importance in projection (VIP) values) in the model inevitably manifested as exogenous compounds, which were primarily enriched in drugs and exogenous organic substances, such as saccharin, lidocaine, and mitozolomide. This phenomenon objectively reflects that chemotherapy induced and consolidated an exposome metabolic fingerprint centered on “drug/exogenous substance clearance stress.” A comparison between the PCBC and BBD groups revealed 993 differentially expressed metabolites. Under the |Log_2_FC| ≥ 1 criterion, the levels of 439 metabolites were significantly increased in the PCBC group, whereas only 8 metabolites were significantly increased in the BBD group. In this cross-group comparison, the differentiation between endogenous and exogenous metabolic signals was particularly pronounced; the metabolites that were present at elevated levels in the BBD group were primarily endogenous metabolites such as steroids and aromatics. In contrast, among the significantly elevated metabolites identified in the PCBC group, in addition to tumor-associated endogenous molecules such as nucleotides, a large proportion was concentrated in drug/exogenous exposures and halogenated organic compounds, such as linezolid (a commonly used adjunct anti-infective agent) and vinclozolin (an environmental exogenous substance). These findings further indicate that, compared with those of the healthy control group, the serum metabolic profiles of breast cancer patients following chemotherapy not only fail to fully recover endogenous homeostasis but are instead deeply imprinted with a persistent “exposure group” signature characterized by high-intensity clinical drug use and exogenous substance exposure. Overall, both the onset of breast cancer and chemotherapy intervention drive extensive and systematic restructuring of the serum metabolic network.

The functions of these metabolites were predicted using the KEGG database to further investigate the inferred functional potential of the microbial communities across the groups (**Figure 8F**). In the comparison between the BBD and BC groups, the prediction results revealed the significant enrichment of pathways related to sphingolipid signaling, purine and other nucleotide metabolism, and autophagy and cell death. These findings suggest a potential regulatory effect of the microbial community on host lipid signaling and energy metabolism during the development of breast cancer. In the comparison between the BC and PCBC groups, pathways such as estrogen signaling, drug metabolism—other enzymes, bile secretion, and riboflavin metabolism were significantly upregulated. Furthermore, comparisons between the PCBC and BBD groups revealed that the predicted potential for purine/nucleotide, carbon, and various amino acid metabolism remained abnormally elevated.

We evaluated the statistical performance of representative differentially abundant metabolites in distinguishing between different clinical states by analyzing receiver operating characteristic (ROC) curves (**Figure 8G**). The results showed that multiple metabolites demonstrated a high discrimination ability (AUC > 0.75) in pairwise comparisons across the three groups. Specifically, the metabolite torsemide achieved an AUC of 0.838 when distinguishing between the BBD and BC groups; cortolone-3-glucuronide had an AUC of 0.780 when distinguishing between the BC and PCBC groups; and trimethylselenonium effectively distinguished between samples from the PCBC and BBD groups, with an AUC of 0.827. Combined with the results of the enrichment analysis, these key metabolites are involved primarily in energy, amino acid, and bile acid/lipid metabolism. Overall, these metabolites with intergroup differences objectively characterize the serum metabolic heterogeneity of different clinical subgroups at the statistical level; however, given that this study employs a single-center observational design, the true clinical translational value of the aforementioned metabolites as biomarkers for breast cancer diagnosis or monitoring the treatment response still requires rigorous validation in future large-scale, multicenter prospective cohorts. ROC curves for responders (R) and nonresponders (NR) in the PCBC cohort were analyzed to further address the need to predict the clinical efficacy of treatment (**Figure 8H**). The predictive value of pharmacokinetic-related metabolites (PK-Ms) was also specifically explored. The results showed that the metabolite preliminarily identified (level 4) as fluoroacetaldehyde displayed excellent discriminatory performance, with an AUC of 0.818 (95% CI: 0.673–0.936) and a VIP of 2.14 (*P* < 0.001). This finding further confirms its key contribution to distinguishing chemotherapy responsiveness. From a biological perspective, fluctuations in fluoroacetaldehyde levels may reflect heterogeneity in fluoropyrimidine metabolism or cellular detoxification pathways among different individuals. Although it was identified as level 4 and requires subsequent structural confirmation, its strong statistical association with the treatment prognosis suggests that fluoroacetaldehyde holds promise as a potential noninvasive biomarker for assessing chemotherapy sensitivity in patients with breast cancer.

**Figure 9:**
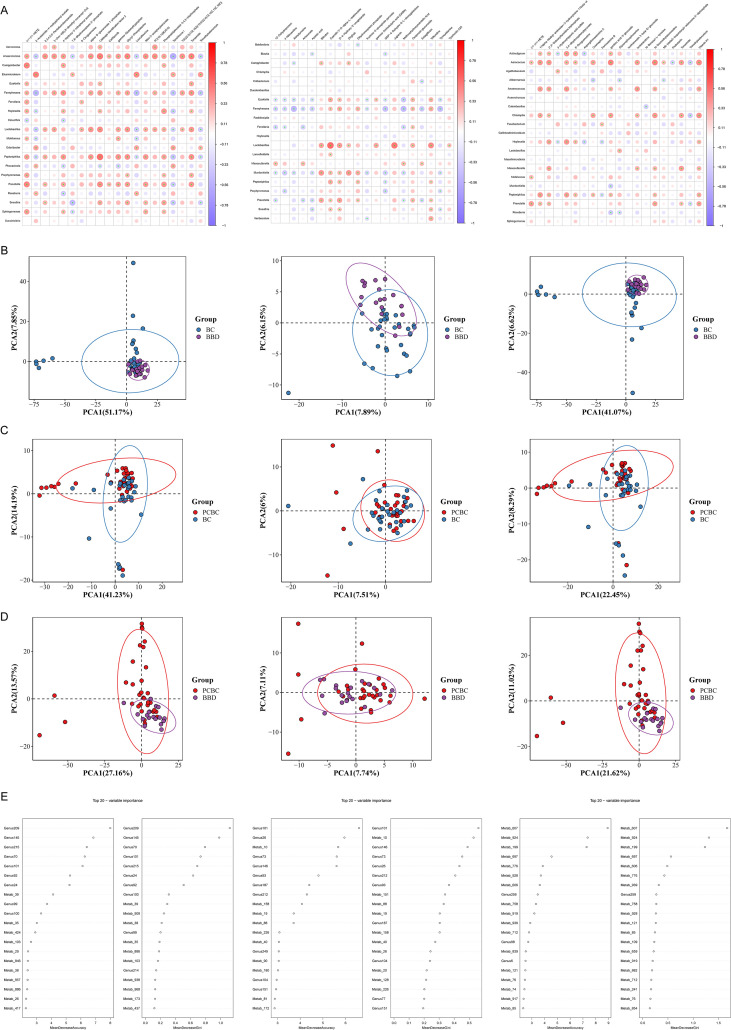
Serum non-targeted metabolomics profiles and their multi-omics integration analysis with the gut microbiome. **(A–C)** Spearman correlation heatmaps of genus-level differential microbiota and serum differential metabolites, showing BBD vs. BC **(A)**, BC vs. PCBC **(B)**, and PCBC vs. BBD **(C)**, respectively. Only significant correlations with |ρ| ≥ 0.3 and p < 0.05 are shown. **(D–F)** Multomics PCA score plots combining gut microbiota and serum metabolites, showing the overall distribution and clustering trends in the integrated metabolomics–microbiome space for the three comparisons: BBD vs. BC **(D)**, BC vs. PCBC **(E)**, and PCBC vs. BBD **(F)**. **(G)** Feature importance ranking plot of the multi-omics random forest model, showing key bacterial genera and differential metabolites distinguishing between groups (ranked by MeanDecreaseAccuracy/MeanDecreaseGini), used to screen for potential biomarkers of breast cancer progression and chemotherapy response. Paired fecal microbiome and serum non-targeted metabolomics samples (BBD = 19, BC = 31, PCBC = 34).

We systematically analyzed the intrinsic relationships between the clinical and pathological characteristics of breast cancer and its metabolic network by constructing a Pearson correlation network of metabolites based on four dimensions: molecular subtype, clinical stage, menopausal status, and age. The results showed that all clinical subgroups exhibited a significant and stable “two-cluster” topological structure characterized by high intracluster synergy (strong positive correlation) and functional antagonism between clusters (negative correlation). At the molecular subtype level (**Supplementary Figures 4A–F**), the most pronounced metabolic differentiation was observed between the HER2+ and TNBC groups (ρ = −0.71), with 4-ethylphenylsulfate and 4-ethoxy ethylbenzoate exhibiting an extremely strong inverse relationship (ρ = −0.93), whereas in the Luminal A and TNBC groups, 3-hydroxydecanoylcarnitine and 2-O-caffeoyltartronic acid exhibited a perfect positive correlation (ρ = 1.00), suggesting extremely tight biochemical regulation within specific subtypes. The clinical staging analysis (**Supplementary Figure 4G**) revealed that in the metabolic networks of patients with early- and late-stage disease, Module A, centered on L-lysine, and Module B, centered on urocanic acid, exhibited persistent antagonism (ρ = −0.24), whereas the negative correlation between methylenetanshinquinone and bialaphos (ρ = −0.65) reflected the disruption of metabolic homeostasis caused by disease progression. Furthermore, menopausal status (**Figure 9H**) and age groups (**Supplementary Figure 4I**) similarly exhibited distinct modular mapping characteristics; the menopause-associated metabolites glycolic acid and oxypurinol demonstrated extremely high synergy (ρ = 0.93), whereas age-related module B exhibited extremely high internal coupling (mean ρ = 0.58), and its core components SM (d18:1/14:0) and 3-methyluridine were significantly negatively correlated (ρ = −0.62).

Notably, although the aforementioned network analysis revealed certain mapping characteristics of age and menopausal status in the metabolic profile, a multivariate logistic regression model was subsequently constructed to exclude their potential interference with the core conclusions as confounding factors. We categorized the signals that were significantly increased after chemotherapy into pharmacokinetic markers (PK-Ms, such as fluoroacetaldehyde) and adjuvant medication and environmental imprints (AM-EIs, such as lidocaine) and re-examined them after adjusting for age and menopausal status. The results revealed that the differences in the distribution of these core metabolic features between groups remained statistically significant (*P* < 0.05). This result provides strong statistical evidence that the metabolic features identified in this study are primarily driven independently by disease biology and chemotherapy intervention, rather than being confounded by baseline demographic characteristics of the patients.

### Serum untargeted metabolomic profile and its association with the gut microbiota

We investigated the associations between the breast disease status and chemotherapy with the gut–metabolic axis by performing a Spearman correlation analysis (|ρ| ≥ 0.30, *P* < 0.05) on samples with paired fecal metagenomic and serum untargeted metabolomic data (BBD n = 19, BC n = 31, and PCBC n = 34). The results showed that most bacterial genera were correlated with various metabolites in clusters, suggesting a potential link between changes in the gut microbiota and host metabolic status (**Figures 9A–C**). In the comparison between the BBD and BC groups (**Figure 9A**), potential opportunistic pathogens enriched in the BC group, such as *Fannyhessea* and *Peptoniphilus*, were generally positively correlated with the levels of inflammatory lipids, nucleic acid oxidative damage markers (8-oxoguanosine), and exogenous-like metabolites, whereas commensal bacteria such as *Roseburia* were associated mainly with the levels of amino acid/peptide metabolites. These findings suggest that under breast cancer conditions, the characteristics of the microbiota–metabolome network shift toward associations with lipid alterations and oxidative damage. In the comparison between the BC and PCBC groups (**Figure 9B**), the abundances of genera such as *Lactobacillus*, which were enriched during the BC phase, were primarily positively correlated with the levels of energy and organic acid metabolites, whereas the abundances of genera such as *Mesosutterella*, which were enriched after chemotherapy, formed new clusters associated with various drug metabolites and bilirubin. These findings indicate that the microbiota–metabolite association patterns after chemotherapy may shift toward exogenous drug metabolism and potential oxidative stress-related factors. In the comparison between the PCBC and BBD groups (**Figure 9C**), opportunistic pathogens such as *Fusobacterium* and *Prevotella*, which were enriched in the PCBC group, were strongly positively correlated with the levels of 8-oxoguanosine and various drug/exogenous chemical metabolites, whereas potentially protective genera such as *Akkermansia* and *Roseburia* were predominantly associated with the levels of polyphenols/phenolic acids and membrane phospholipid-related metabolites. This result further reflects that the metabolic profile is characterized by an increased metabolic burden of exogenous substances in the postchemotherapy phase. In summary, the heatmaps of the three groups reveal characteristic microbiota–metabolite association patterns under different disease and treatment conditions.

Based on microbial community and differentially abundant metabolite data at the genus level, we comprehensively analyzed the changes in metabolic and microbiome characteristics during the progression of benign breast lesions to breast cancer and during the course of chemotherapy by performing PCA on metabolite, microbial community, and combined metabolite–microbiome data for the three groups: BBD vs. BC, BC vs. PCBC, and PCBC vs. BBD groups. PERMANOVA was used to evaluate intergroup differences. In the comparison between the BBD and BC groups, the metabolite PCA plot showed clear separation between the two groups, with the BC group exhibiting a more dispersed distribution; the genus-level PCA plot showed a certain degree of separation, and the combined PCA plot displayed significant separation. PERMANOVA revealed significant differences in the metabolic profiles (*F* = 25.02, *P* = 0.001), microbial community structure (*F* = 6.83, *P* = 0.001), and combined profiles (*F* = 25.48, *P* = 0.001). In the comparison between the BC and PCBC groups, both the metabolite PCA and combined PCA plots showed clear separation between the two groups, whereas the bacterial genus PCA plot exhibited a certain degree of separation; PERMANOVA revealed significant differences in the metabolic profile (*F* = 20.47, *P* = 0.001), microbial community structure (*F* = 2.97, *P* = 0.014), and combined profile (*F* = 16.29, *P* = 0.001). In the comparison between the PCBC and BBD groups, both the metabolite PCA and the combined PCA plots showed clear separation between the two groups, whereas the bacterial genus PCA plot exhibited a certain trend toward separation; PERMANOVA revealed significant differences in the metabolic profile (*F* = 9.98, *P* = 0.001), microbial community structure (*F* = 2.01, *P* = 0.045), and combined profile (*F* = 11.02, *P* = 0.001). These results indicate that significant metabolic and microbial differences exist between patients with breast cancer and those with benign lesions. Chemotherapy intervention can partially restore metabolic and microbial characteristics toward a benign state, but these characteristics have not yet fully returned to normal levels.

**Figure 10:**
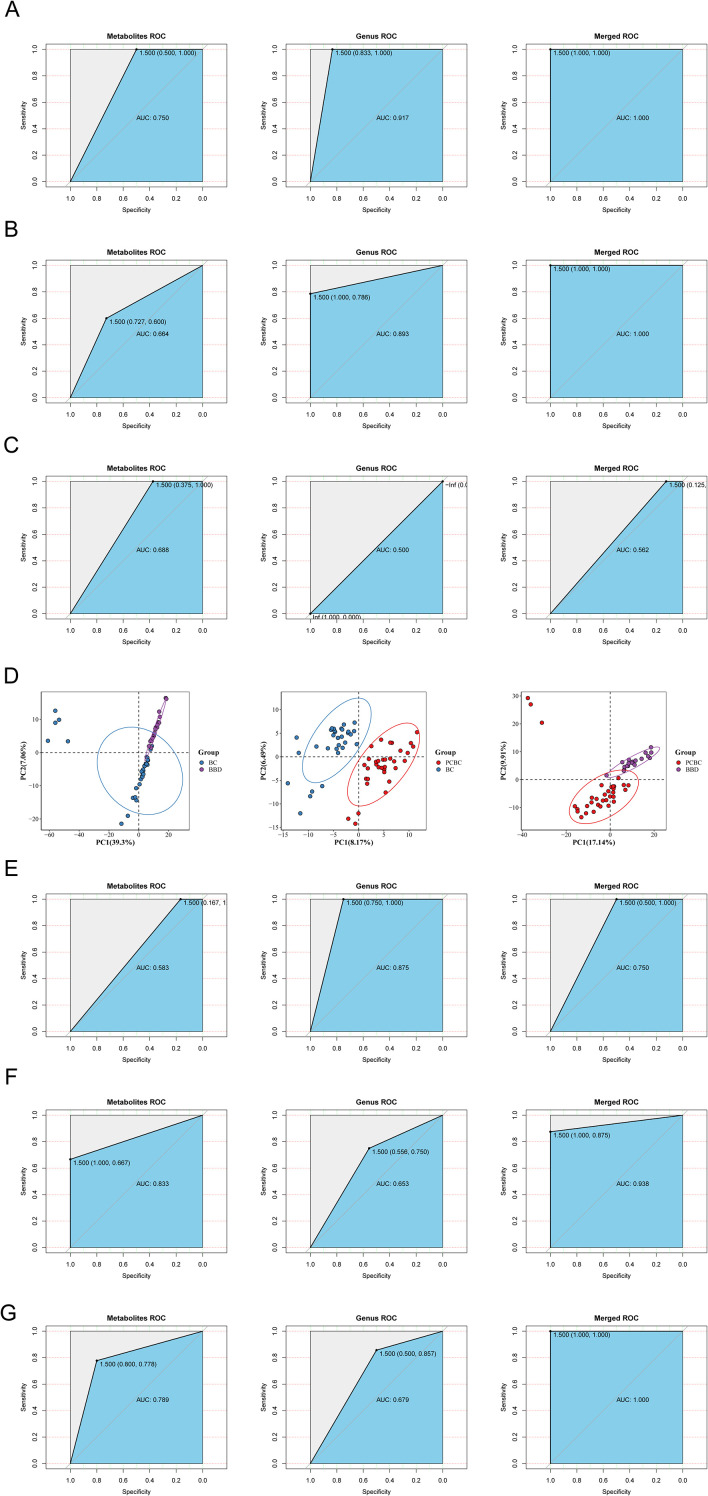
Comparison of classification performance between Random Forest and PLS-DA multi-omics models. **(A–C)** ROC curves for the microbiome model, metabolomics model, and combined model based on Random Forest when distinguishing **(A)** BBD vs. BC, **(B)** BC vs. PCBC, and **(C)** PCBC vs. BBD. The combined model achieved an AUC of 1.000 for BBD vs. BC and BC vs. PCBC, significantly outperforming single-omics models. **(D)** Partial Least Squares Discriminant Analysis (PLS-DA) plot. **(E–G)** ROC curves for the microbiome, metabolome, and combined models based on PLS-DA. **(E)** The microbiome model exhibited the highest AUC in BBD vs. BC; **(F)** the metabolomics and combined models demonstrated the strongest discriminatory ability in BC vs. PCBC and **(G)** PCBC vs. BBD, suggesting that microbiome changes predominate during the onset of breast cancer, while metabolic remodeling predominates during and after chemotherapy. Analysis of fecal microbiome and serum non-targeted metabolomics samples (BBD n=19, BC n=31, PCBC n=34).

In this study, genus-level microbial abundance data were integrated with differentially abundant metabolite data to construct a random forest classification model for identifying key biomarkers that distinguish between different groups. The discriminatory performance of the model was validated using ROC curves, and the contribution of each variable to group differentiation was assessed based on the MDA and the MDG (**Figure 10G**). In the discriminant models for the BBD and BC groups, a series of feature variables with high contributions were identified. With respect to microorganisms, the most significant discriminatory capabilities were observed for genera such as *Peptoniphilus* (Genus 209), *Lactobacillus* (Genus 145), *Corynebacterium* (Genus 70), *Finegoldia* (Genus 101), and *Prevotella* (Genus 215). With respect to metabolites, beta-citryl-L-glutamic acid (Metab_39), L-lysine (Metab_103), citric acid (Metab_35), and trans-aconitic acid (Metab_38) exhibited the highest variable importance. These results indicate that the state differences between breast cancer and benign lesions are primarily reflected in specific microbial community structures and characteristic alterations in energy and amino acid metabolic pathways. Regarding the comparison between the BC group and the PCBC group, the key distinguishing features screened by the model at the microbial genus level were contributions from *Fannyhessea* (Genus 101), *Anaerococcus* (Genus 26), *Corynebacterium* (Genus 73), and *Lactobacillus* (Genus146). With respect to metabolites, thiourea (Metab_10), mesaconic acid (Metab_19), and arabinonic acid (Metab_88) displayed the most prominent discriminatory ability. The differential distribution of these features reflects that, following chemotherapy intervention, the patient’s host metabolic profile and specific microbial abundance significantly differed from those observed preoperatively. In the comparative analysis of the PCBC group and the BBD group, the model indicated that metabolite features played a dominant role in distinguishing between the groups. Representative differentially expressed metabolites included N6, N6, N6-trimethyl-L-lysine (Metab_607), sulfate (Metab_199), choline (Metab_924) and L-glutamine (Metab_697). Additionally, microbial genera such as *Ureaplasma* (Genus 259), *Lactobacillus* (Genus 146), and *Acinetobacter* (Genus 6) also had certain discriminatory value. These results indicate that the metabolic profiles of patients following chemotherapy still differ significantly from those of patients with benign disease and that certain microbial characteristics retain important reference value in distinguishing between these two groups.

Two complementary models, random forest and PLS-DA, were comprehensively employed in this study. The former focuses on evaluating the discriminatory power of multiomics features, while the latter aims to reveal the contributions of different features to sample clustering; together, they were used to systematically evaluate the classification performance of multiomics data. A ROC curve analysis based on a random forest model revealed that the multiomics integrated model demonstrated good discriminatory power in distinguishing breast disease status from chemotherapy intervention. In the comparisons between the BBD and BC groups (**Figure 10A**) and between the BC and PCBC groups (**Figure 10B**), the combined model displayed the optimal discriminatory performance (AUC of 1.000, with both sensitivity and specificity of 1.000), significantly outperforming the single-genus models (AUC of 0.917 for the comparison of the BBD and BC groups and 0.893 for the comparison of the BC and PCBC groups) and metabolite models (AUC of 0.688 for the comparison of the BBD and BC groups and 0.656 for the comparison of the BC and PCBC groups), indicating that multiomics integration can better distinguish between benign and malignant states as well as samples collected before and after chemotherapy. However, in the comparison between the PCBC and BBD groups (**Figure 10C**), the bacterial genus model (AUC = 0.500) and the combined model (AUC = 0.562) demonstrated a limited discriminatory ability, while the metabolite model (AUC = 0.667) performed relatively better, suggesting that the intergroup differences between postchemotherapy patients and the benign population are primarily reflected at the metabolic level. Based on the PLS-DA model, we further evaluated the effects of breast disease progression and chemotherapy intervention on relevant omics features. The results showed that the omics features exhibited stage-specific changes accompanying disease progression and chemotherapy intervention. In the multivariate analysis, the PCBC group as a whole fell between the BBD and BC groups (**Figure 10D**), suggesting that although chemotherapy intervention modulated omics features, the overall metabolic profiles of patients still differed from those of the benign population. Furthermore, PCA indicated that the three groups of samples exhibited a certain degree of separation at the single-metabolite level, but overlap persisted in the boundary regions. ROC curves were additionally plotted based on the PLS-DA model to compare the discriminatory performance of each omics approach. In the comparison of the BBD and BC groups, the AUC of the microbiome model was 0.875, which was higher than that of the metabolomics and combined models, suggesting that differences in microbial abundance were more pronounced in the comparison between benign and malignant states (**Figure 10E**). In the comparison of the BC and PCBC groups, the metabolomic model achieved an AUC of 0.944, outperforming both the microbiome model and the combined model, indicating that changes in host metabolic pathways before and after chemotherapy were more pronounced (**Figure 10F**). In the comparison of the PCBC and BBD groups, both the metabolomic and the multiomics combined models achieved an AUC of 1.000 (**Figure 10G**), reflecting significant systemic differences in the metabolic status between the postchemotherapy group and the benign group. Taken together, these results indicate that a multiomics integrated strategy helps to more comprehensively reflect the characteristic changes at different stages of breast disease. Specifically, differences in the gut microbiota are primarily evident in comparisons between benign and malignant breast states, whereas changes in the metabolic profile play a dominant role in reflecting the effects of chemotherapy intervention and its subsequent effects.

## Discussion

By integrating gut microbiome and serum metabolomic data, the dynamic remodeling of the host microbiome and metabolic networks were systematically mapped throughout disease progression from BBD to BC and PCBC in this study. The results not only revealed significant alterations in the gut microbiota and systemic metabolic profiles in breast cancer patients but also provided a preliminary map of the interaction network between the gut microbiota and serum metabolites through a multiomics correlation analysis, providing observational evidence for the “gut–breast axis” hypothesis from the perspectives of both microbial ecology and host metabolism. First, we analyzed the gut microbiota characteristics of breast cancer patients using both cross-sectional and longitudinal data. The cross-sectional analysis revealed that while the Shannon and Simpson indices differed among the BBD, BC, and PCBC groups, the Chao and ACE indices, which represent species richness, and the community structure indicated by NMDS remained generally stable; similarly, a longitudinal paired analysis showed no significant changes in α diversity, suggesting that the gut microbiota exhibits a certain degree of resilience under the stress of chemotherapy. According to the perspective proposed by Moya and Ferrer (25), this stability may be related to functional redundancy within the microbiome. In this study, although the abundance of the key butyrate-producing bacterium *Faecalibacterium* decreased in the BC group and the postchemotherapy SPCBC group, bacterial communities with similar metabolic functions, such as *Roseburia*, *Anaerostipe*s, and *Blautia*, remained stable or showed compensatory increases in abundance, suggesting that the core metabolic network may maintain overall homeostasis through niche compensation. Despite a relatively stable overall structure, different groups still exhibited heterogeneity in the composition of specific microbial communities. Cross-sectional comparisons revealed that *Bacteroidota* was relatively enriched in the PCBC group, whereas *Fusobacteriota* was more abundant in the BC group; longitudinal follow-up further demonstrated that the abundance of *Faecalibacterium* decreased significantly after chemotherapy, whereas the abundances of potential opportunistic pathogens such as *Phocaeicola* and *Escherichia* increased. Overall, the conclusions drawn from longitudinal paired samples and cross-sectional comparisons are largely consistent, namely, that chemotherapy primarily affects the relative abundance of specific bacterial groups without causing a significant destabilization of the overall community structure. However, the specific bacterial groups identified as differentially affected do not fully overlap between the two approaches. This discrepancy is not contradictory; rather, it likely reflects different informational dimensions at the study design level. Specifically, the cross-sectional analysis primarily reveals population-level associations between different clinical states and is susceptible to individual baseline differences, whereas the longitudinal analysis controls for interindividual heterogeneity and better reflects temporal changes associated with chemotherapy exposure. The PCBC group exhibited greater intragroup heterogeneity based on the weighted UniFrac distance, further suggesting differences in the microbiological response to chemotherapy among individuals. However, this study was primarily based on a single-center cross-sectional design, with only 24 of the 295 participants providing paired longitudinal samples. Previous studies have shown that host factors such as diet, lifestyle, age, and concomitant medication can significantly influence the gut microbiota composition (26). In the absence of more thorough control for confounding factors, some between-group differences may reflect individual characteristics rather than the disease or treatment itself. Furthermore, as this study was observational, the current results support only an association between the gut microbiota and clinical status of patients with breast cancer; they are insufficient to infer causality and cannot be used to directly regard the associated microbial profiles as reliable biomarkers for diagnostic stratification or treatment monitoring. Moreover, data based on 16S rRNA sequencing cannot be used to directly assess microbial metabolic activity. Therefore, the interpretation of functional redundancy presented in this paper remains speculative and is based on changes in taxonomic abundance. Future studies will need to incorporate multicenter, large-sample longitudinal cohorts and integrate multiomics analyses, such as metagenomics and metabolomics, to more thoroughly exclude confounding factors and validate the mechanistic significance and clinical translational value of these microbial changes (27). In summary, the overall structure of the gut microbiota in patients with breast cancer undergoing chemotherapy is somewhat stable, but the composition of specific microbial communities may undergo selective remodeling. These findings support an association between the gut microbiome and the context of breast cancer treatment. However, its potential as a therapeutic target or monitoring biomarker requires further rigorous validation.

We analyzed the relationships between the gut microbiota of breast cancer patients and their clinical and pathological characteristics, as well as their response to chemotherapy. Patients with different molecular subtypes exhibited relatively distinct microbial profiles. Before and after chemotherapy, the differences in β diversity among molecular subtypes were statistically significant, indicating an association between intrinsic tumor biological differences and changes in the gut microbiome; moreover, this association did not completely disappear following chemotherapy. Previous studies have shown that the gut and tumor-associated microbial composition in breast cancer patients is associated with the ER, PR, and HER2 status and that different molecular subtypes may exhibit distinct metabolic backgrounds and immune states, providing a basis for the findings of this study (28, 29). From a clinical perspective, these findings suggest that the gut microbiota reflects not only the body’s overall metabolic and inflammatory statuses but also, to some extent, the molecular heterogeneity of breast cancer. Furthermore, we observed more pronounced alterations in the gut microbiota of patients with advanced-stage breast cancer, which were manifested as changes in the relative abundances of certain bacterial genera associated with inflammation, mucosal homeostasis, and metabolic function. In patients with advanced-stage tumors, the abundances of certain short-chain fatty acid-producing bacterial communities were reduced, while the proportions of opportunistic pathogens or facultative anaerobes increased. This trend is consistent with the understanding of an increased tumor burden, systemic inflammatory activation, and impaired intestinal barrier homeostasis (30, 31). Previous studies have suggested that alterations of the gut microbiota may contribute to breast cancer progression through immune regulation, estrogen cycling, bile acid metabolism, and the release of inflammatory mediators (32, 33). Therefore, the stage-related microbial differences observed in this study may reflect systemic alterations in the host metabolic–immune–microbiome network against the backdrop of tumor progression.

In this study, LEfSe indicated that the microbial differences between the BC and PCBC groups were not uniformly distributed but were significantly dependent on the molecular subtype and clinical stage, suggesting that chemotherapy-related changes in the microbiome are influenced by the intrinsic biological characteristics of the tumor (34). At the molecular subtyping level, patients with the Luminal A/B subtype exhibited a shift from a predominantly anaerobic/fermentative microbiota toward taxonomic groups such as *Gammaproteobacteria* and *Bacteroidia* following chemotherapy, consistent with previous reports that chemotherapy disrupts the intestinal redox state and niche structure (32, 35). In contrast, *Actinomycetota–Atopobiaceae*-related characteristics were more stable in patients with the HER2+ subtype (36), whereas the abundance of the inflammation-associated taxon *Fusobacteriota* in the TNBC group decreased significantly after chemotherapy, suggesting that the microbiome of patients with this subtype is more sensitive to chemotherapy exposure (37). Stratification by clinical stage revealed that patients with advanced-stage disease exhibited a significant trend toward a shift from anaerobic fermentative types (e.g., *Clostridia*) to facultative anaerobic or aerobic types following chemotherapy, whereas differences among early-stage patient groups were minimal. These findings suggest that treatment-related microbiome alterations are not yet prominent when the tumor burden is low and that the chemotherapy-induced disruption of the microbiome intensifies as the disease progresses. Within the BC cohort, different subtypes exhibited unique bacterial genus enrichment patterns; for example, the Luminal A group was enriched with *Sutterella*, and the Luminal B group was enriched with *Parasutterella*, which may reflect differences in metabolic or mucosal immune backgrounds among subtypes (34, 38). These findings further support the notion that the gut microbiota functions as a “microbiome phenotype” associated with subtyping rather than as a direct pathogenic factor. Within the PCBC group, nonresponders (NRs) showed significant enrichment of bacterial communities such as *Collinsella*, which are associated with pro-inflammatory states and barrier damage (35, 39), whereas responders (R) were characterized by *Pseudomonas* enrichment. This treatment response pattern is not simply a matter of “probiotic” fluctuations but may involve the regulation of chemotherapy sensitivity by bacterial components through TLR-related pathways (36). In summary, the gut microbiota is closely associated with breast cancer subtypes, stage, and treatment response, with this association being particularly pronounced in patients with advanced and highly aggressive subtypes.

Using a nontargeted metabolomic approach, we systematically characterized the metabolic remodeling trajectories underlying the progression of breast diseases and the effects of chemotherapy. During the progression from benign breast disease (BBD) to breast cancer (BC), the significant enrichment of the purine metabolism and autophagy pathways reflects the adaptive regulation of energy and environmental stress by tumor cells to meet their proliferative demands (40–42). In contrast, the transition from BC to PCBC primarily involves the activation of estrogen signaling and exogenous substance degradation pathways, reflecting the body’s systemic metabolic response to the chemotherapy burden. Notably, based on the results from PERMDISP analysis, the PCBC group exhibited significantly greater intragroup heterogeneity than the other groups (*F* = 4.40, *P* = 0.013; see **Figure 1G**). According to the “Anna Karenina Principle,” this increase in heterogeneity suggests a stochastic shift in the microbiome and metabolic homeostasis under intense therapeutic stress, driven jointly by the heterogeneity in the treatment response and the individual exposome (13, 43).

When interpreting differences before and after chemotherapy, distinguishing between “disease-specific biological effects” and “exogenous exposure effects” is essential. The signals identified in this study, such as those associated with lidocaine, linezolid, and vinclozolin, should be regarded as a true reflection of changes in the exposome during chemotherapy. Interestingly, the “inferred functional potential” related to exogenous substance degradation in the gut microbiome is significantly coupled with the serum levels of the aforementioned exogenous metabolites, suggesting that the heterogeneity observed in the PCBC group is essentially a dual manifestation of “residual endogenous tumor metabolic abnormalities” and “host–microbiota collaborative responses to the burden of drug clearance” (10, 44). Therefore, while exogenous interference should be excluded when screening for diagnostic biomarkers in the future, these characteristics and their interactions with the microbiota hold potential monitoring value when assessing chemotherapy tolerance and the metabolic response. Although these exogenous compounds are not suitable as disease-specific biomarkers, their coupling with the gut microbiota’s exogenous metabolite degradation function provides a valuable window into understanding host–microbiota cometabolism during chemotherapy. After exogenous signals were further removed, a comparison between the PCBC and BBD groups revealed that even after the completion of standard chemotherapy, certain purine/nucleotide and energy metabolism markers in patients had not yet returned to the levels in patients with benign disease. These findings suggest that breast cancer and its intensive treatment leave a persistent “metabolic imprint” in the body, which may be associated with the risk of recurrence, long-term metabolic complications, and delayed fatigue (41, 45). The identification of this metabolic imprint not only reinforces its clinical value as a tumor marker but also provides a new perspective for the long-term management of patients after chemotherapy.

Using a univariate strategy with an AUC threshold of >0.7, we identified several representative metabolic signatures and preliminarily characterized metabolic remodeling at different stages of breast cancer. The ROC curve analysis showed that torsemide had high discriminatory power in identifying the early benign-to-malignant transformation (AUC = 0.838); moreover, cortolone-3-glucuronide effectively reflected chemotherapy-induced stress states and fluctuations in glucocorticoid metabolism, whereas trimethylselenonium may characterize postchemotherapy redox residues. Furthermore, the significant enrichment of fluoroacetaldehyde in patients who responded to chemotherapy is highly biologically plausible as it is a key intermediate in the catabolism of fluoropyrimidine drugs (such as 5-FU), and differences in its abundance may reflect unique drug processing mechanisms within the tumor, suggesting a better clinical prognosis. Although this analysis is currently limited by the lack of commercially available reference standards, the identification of fluoroacetaldehyde is at MSI level 4. We were unable to collect plasma samples from a longitudinal cohort of 24 patients for validation, but its strong statistical significance and metabolic logic make it a promising candidate biomarker for assessing the treatment response (14). However, in this study, only the diagnostic performance of a single metabolite was evaluated. Although the AUC of some biomarkers approached 0.8, a single feature cannot comprehensively capture the highly complex metabolic heterogeneity during breast cancer progression. Theoretically, constructing a multimetabolite diagnostic panel with biological complementarity is the standard approach to increasing its clinical utility. Due to the design and sample size limitations of this exploratory study, we have not yet established a robust diagnostic panel, which constitutes a limitation of this study. In the future, a multimarker panel should be developed using the data from larger prospective cohorts through machine learning modeling, rigorous chemical characterization, and independent validation. Concurrently, multidimensional data such as transcriptomic and proteomic data must be integrated to elucidate the upstream mechanisms driving these metabolic shifts, thereby truly enhancing the clinical translational value of metabolic biomarkers in early detection, treatment response monitoring, and prognostic assessment.

By integrating gut metagenomics with serum nontargeted metabolomics, we systematically characterized the dynamic landscape of the “microbiota–host metabolism–breast tumor” axis during disease progression and upon chemotherapy intervention. First, the progression from BBD to BC is accompanied by a systemic shift in the microbiota–metabolic network. We observed that the positive correlations between beneficial commensal bacteria, such as *Lactobacillus* and *Roseburia*, and amino acid and energy metabolism gradually weakened, whereas the associations between opportunistic pathogens, such as *Campylobacter* and *Peptoniphilus*, and proinflammatory and oxidative stress metabolites were significantly strengthened. These findings provide direct multiomics evidence for the role of the “gut–breast axis” in the regulation of inflammation and metabolic reprogramming in breast cancer (46–48). Upon entering the chemotherapy phase, this coupling pattern further shifts toward being dominated by exogenous drug metabolism and persistent microbiome stress. We identified significant associations between specific bacterial genera, such as *Blautia* and *Porphyromonas*, and metabolites of chemotherapeutic drugs, suggesting that the gut microbiota not only participates in drug metabolism but also may influence patients’ long-term outcomes by inducing metabolic imbalances. These findings are highly consistent with recent conclusions from prospective studies on the roles of the microbiota in modulating chemotherapy toxicity and patients’ prognosis (41, 49, 50). Second, multiomics integrated modeling has demonstrated significant advantages in disease classification and status differentiation. Compared with single omics approaches, random forest models indicate that the combination of “microbiome + metabolome” features exhibits superior performance in terms of the AUCs for distinguishing between benign and malignant transformations and pre- and postchemotherapy states. Particularly before and after chemotherapy, the classification contribution of metabolic reprogramming signals is more pronounced, identifying a cluster of reproducible microbial–metabolite “fingerprint” features, which provides important insights for the development of clinically stable biomarkers across cohorts (51–53). Notably, although the combined models for BBD vs. BC and BC vs. PCBC achieved an AUC of 1.000 in this study, a risk of overfitting exists given the small sample size of 84 patients and the high dimensionality of the multiomics data. Therefore, the aforementioned AUC values should be interpreted as exploratory findings rather than accurate estimates of the model’s true generalizability. The robustness of the model needs to be re-evaluated using an independent validation cohort (54, 55).

In terms of functional interpretation, this study utilized 16S rRNA sequencing combined with PICRUSt2 to predict microbial metabolic pathways. Notably, such analyses represent only the “inferred functional potential” of the microbiota rather than directly measuring biological activity (41). In particular, for the pathways identified in this study, including xenobiotic metabolism, steroid hormone biosynthesis, and cytochrome P450 drug metabolism, the precise metabolic fluxes still require confirmation through future metagenomic or metatranscriptomic studies. Nevertheless, the multiomics association analysis revealed a core evolutionary pattern: from benign lesions through breast cancer to the postchemotherapy phase, the association between the microbiota and host metabolism tended to shift from energy/amino acid metabolism toward lipid alterations, xenobiotic metabolism, and oxidative stress. This shift provides multiomics-level insights for understanding breast cancer and chemotherapy-related gut microbial alterations (41).

Although this study provides extensive multiomics evidence, the following limitations remain. First, as a single-center observational study, this study primarily revealed associations between the microbiome characteristics and tumor status/chemotherapy exposure; it is insufficient to infer definitive causal relationships. Its potential value in diagnostic stratification or treatment monitoring for breast cancer still needs to be validated in future large-scale prospective cohorts (39). Second, we employed only univariate screening methods (AUC ≈ 0.8) when evaluating the diagnostic performance of metabolic biomarkers. While this approach aids in identifying specific targets, constructing a diagnostic panel comprising multiple complementary metabolites is the standard practice for significantly improving clinical accuracy. Regrettably, due to limitations in our current data analysis workflow, we were unable to construct a robust multimetabolite diagnostic model, which represents an important direction for future research. Furthermore, due to limitations in the initial study design, we were unable to collect plasma samples from the 24 patients included in the initial longitudinal cohort, which prevented us from simultaneously analyzing the dynamic changes in systemic metabolism during chemotherapy; this issue should be addressed in future studies.

## Data Availability

The original contributions presented in the study are publicly available. This data can be found here: National Center for Biotechnology Information (NCBI) under BioProject accession number PRJNA1398789, and MetaboLights under accession number MTBLS13634.

## Supplementary material

Supplementary Figure 1 **(A–C) (A)** Differences in gut microbial functions between BBD and BC based on KEGG level 3 pathways. **(B)** Comparison of KEGG level 3 functional pathways before and after chemotherapy (BC vs. PCBC). **(C)** KEGG level 3 pathway differences between PCBC and BBD (Wilcoxon test, *P* < 0.05, **P* < 0.01).

Supplementary Figure 2 Bar plots of the relative abundances of KEGG level 3 functional categories.

Supplementary Figure 3 No significant associations were observed between breast cancer molecular subtypes, clinical stages, and gut microbiota α-diversity (ACE, Chao, Shannon, and Simpson indices) (*P* > 0.05). **(A–D)** α-diversity by molecular subtype in the BC group; **(E–H)** α-diversity by molecular subtype in the PCBC group; **(I–L)** α-diversity by clinical stage in the BC group and the PCBC group.

Supplementary Figure 4 Metabolite correlation network analysis based on different clinical and pathological characteristics **(A–F)** Pearson correlation networks of metabolites within different molecular subtypes (Luminal A, Luminal B, HER2^+^, TNBC). **(G)** Comparison of metabolite correlation networks between early-stage (Stages I–II) and advanced-stage (Stages III–IV) patients. **(H)** Metabolite correlation networks for patients with different menopausal statuses. **(I)** Metabolite correlation networks for patients in different age groups. All networks are constructed based on Pearson correlation coefficients, illustrating the common characteristics of positive correlations (synergy) within modules and negative correlations (antagonism) between modules.
