# Supplementary figures and images for: Dynamic changes and clinical significance of the gut microbiota and serum metabolites in breast cancer onset, progression and chemotherapy intervention
Source: Front Oncol. 2026 May 14;16:1795317. doi: 10.3389/fonc.2026.1795317 (PMC13215807; doi:10.3389/fonc.2026.1795317)

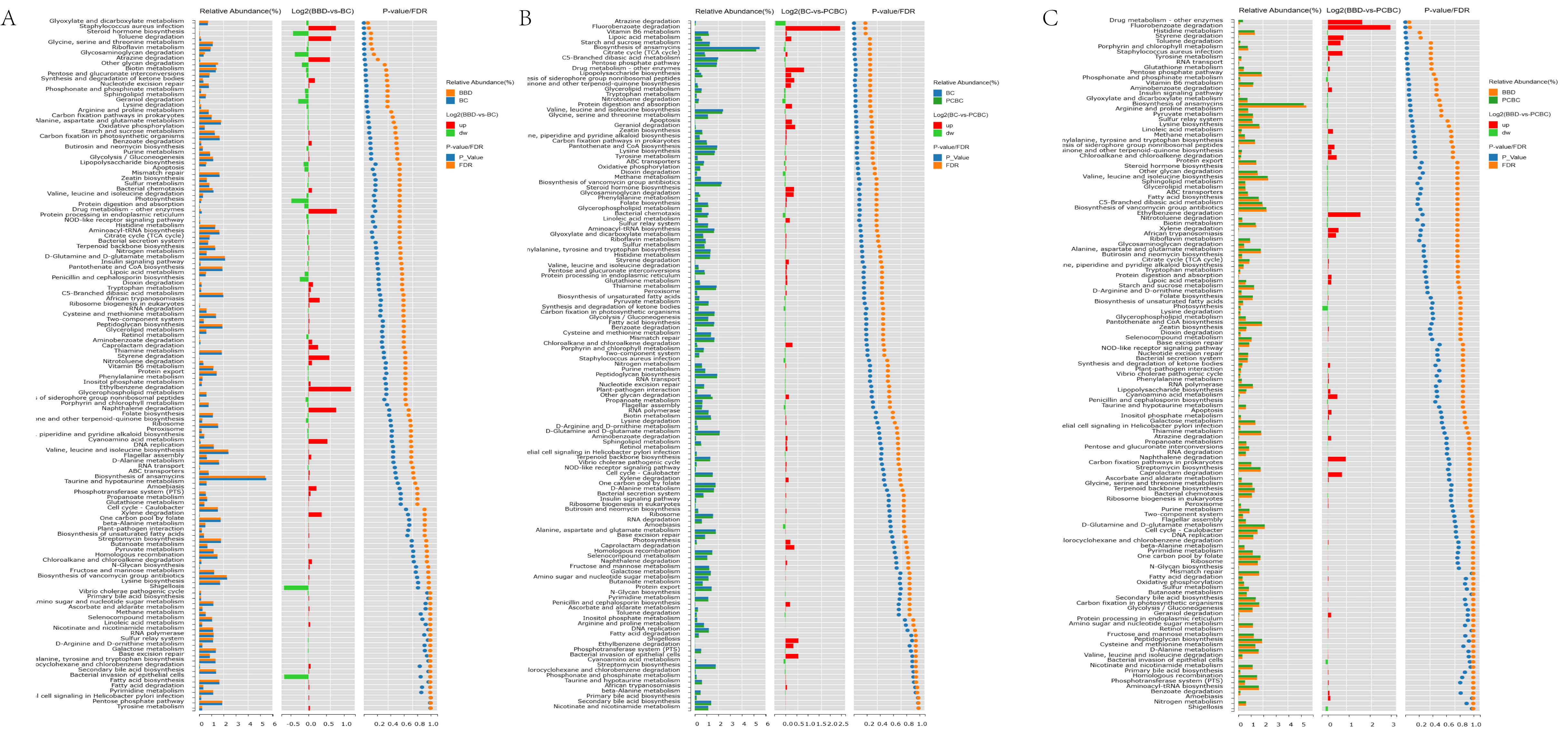

Supplement: Supplementary file 1 [file Image1.tif]

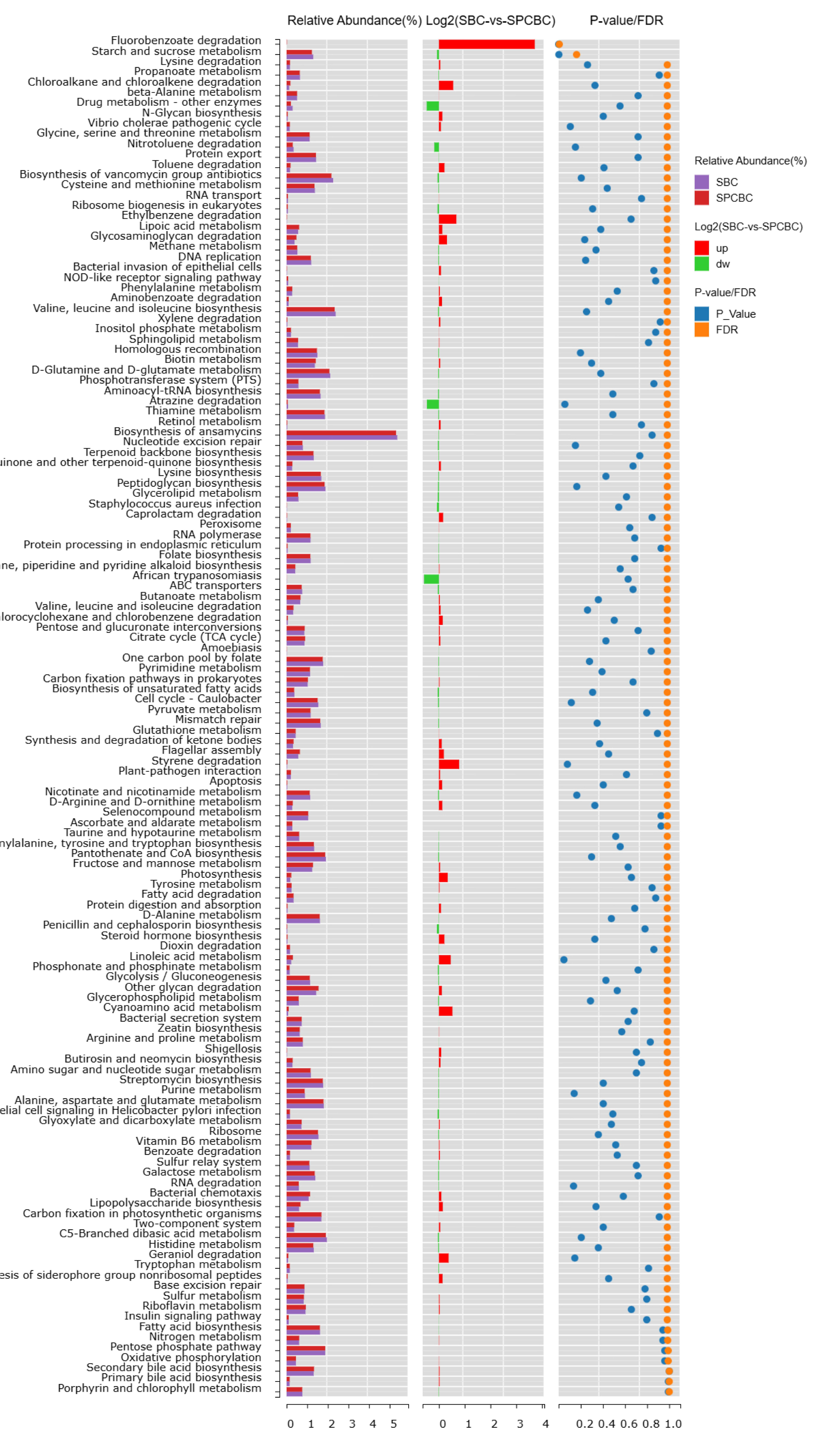

Supplement: Supplementary file 2 [file Image2.tif]

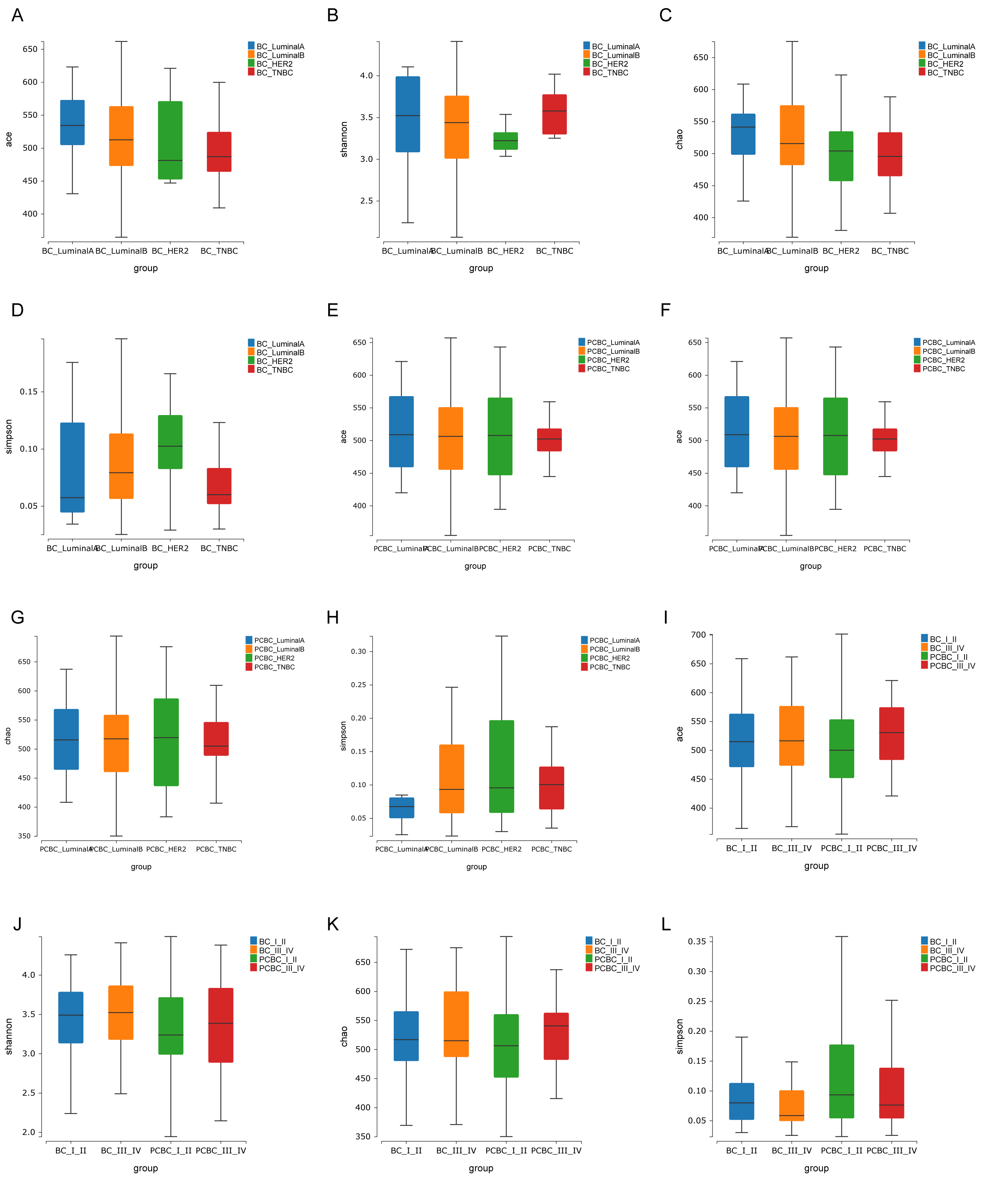

Supplement: Supplementary file 3 [file Image3.tif]

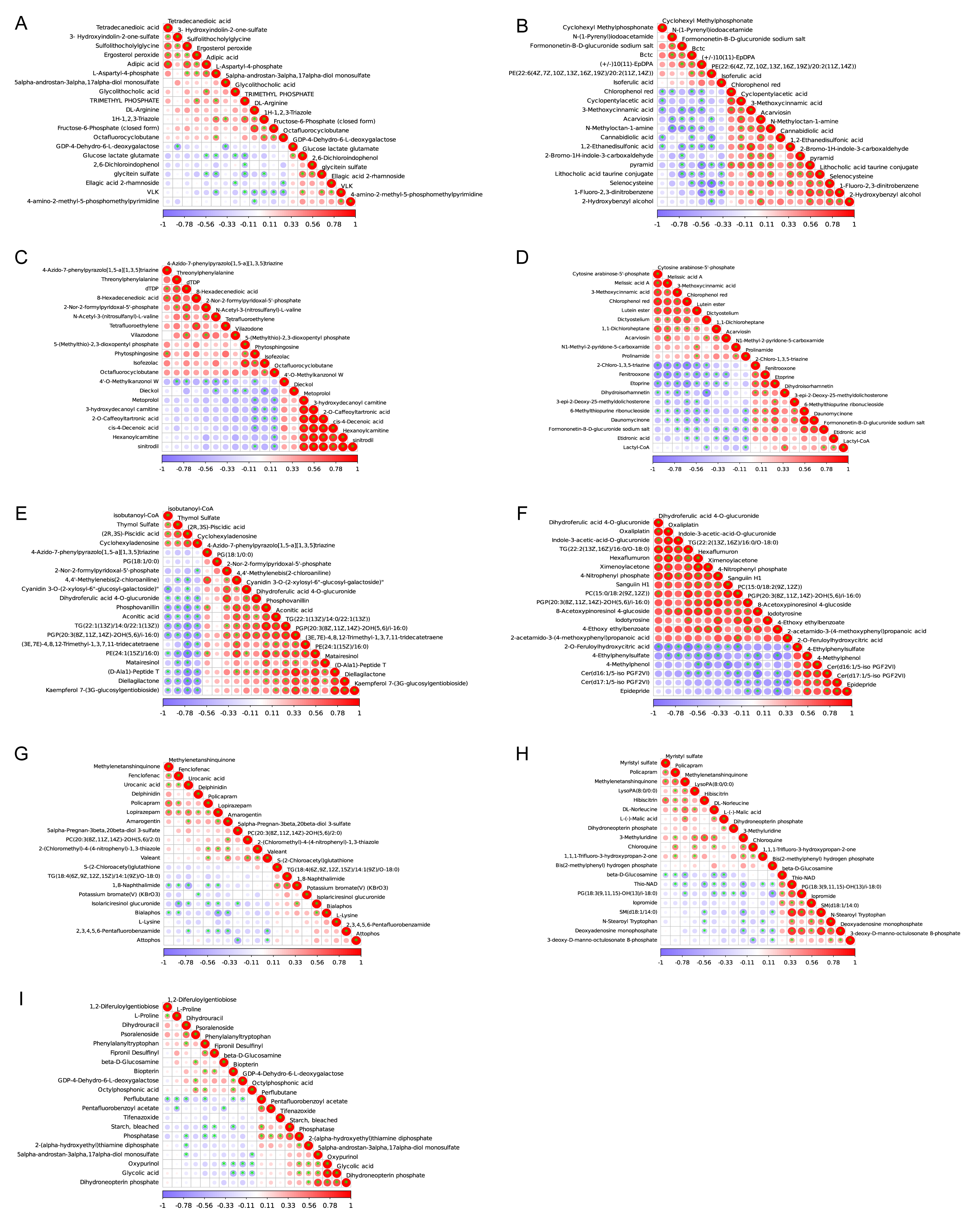

Supplement: Supplementary file 4 [file Image4.tif]
