# Supplementary material for: Dynamic changes and clinical significance of the gut microbiota and serum metabolites in breast cancer onset, progression and chemotherapy intervention
Source: Front Oncol. 2026 May 14;16:1795317. doi: 10.3389/fonc.2026.1795317 (PMC13215807; doi:10.3389/fonc.2026.1795317)
Supplement: Supplementary file 5 [file Table1.docx]

**Supplementary Table 1**.Multivariate logistic regression analysis incorporating clinical confounders and core multi-omics biomarkers

| **Comparison** | **Variables in Model** | **OR (95% CI)** | ***P*value** |
| --- | --- | --- | --- |
| Stage of the disease | Clinical confounding factors |  |  |
| BC vs. BBD | Age | 1.04 (0.99–1.09) | 0.120 |
| (0=BBD, 1=BC) | Menopausal status (postmenopausal vs. premenopausal) | 2.31 (1.02–5.24) | 0.045 |
|  | Omics Signatures |  |  |
|  | Faecalibacterium | 0.78 (0.62–0.98) | 0.035 |
| Chemotherapy intervention phase | Clinical confounding factors |  |  |
| PCBC vs. BC | Age | 1.01 (0.96–1.05) | 0.650 |
| (0=BC, 1=PCBC) | Menopausal status (postmenopausal vs. premenopausal) | 1.15 (0.62–2.13) | 0.580 |
|  | Omics Signatures |  |  |
|  | Phocaeicola | 1.85 (1.12–3.05) | 0.018 |
|  | Lidocaine | 4.25 (1.50–12.1) | 0.007 |
| Residual effects after chemotherapy | Clinical confounding factors |  |  |
| PCBC vs. BBD | Age | 1.02 (0.97–1.07) | 0.380 |
| (0=BBD, 1=PCBC) | Menopausal status (postmenopausal vs. premenopausal) | 1.89 (0.85–4.21) | 0.120 |
|  | Omics Signatures |  |  |
|  | Enterococcus | 1.45 (1.02–2.06) | 0.039 |
|  | Mitozolomide | 2.85 (1.40–5.80) | 0.004 |
